# Reduction in hepatic secondary bile acids caused by short-term antibiotic-induced dysbiosis decreases mouse serum glucose and triglyceride levels

**DOI:** 10.1038/s41598-018-19545-1

**Published:** 2018-01-19

**Authors:** Takuya Kuno, Mio Hirayama-Kurogi, Shingo Ito, Sumio Ohtsuki

**Affiliations:** 1 Department of Pharmaceutical Microbiology, Graduate School of Pharmaceutical Sciences, Kumamoto University, 5-1 Oe-honmachi, Chuo-ku, Kumamoto, 862-0973 Japan; 2 Department of Drug Metabolism and Pharmacokinetics, Nonclinical Research Center, Tokushima Research Institute, Otsuka Pharmaceutical Co., Ltd., 463-10 Kagasuno, Kawauchi-cho, Tokushima, 771-0192 Japan; 3 Department of Pharmaceutical Microbiology, Faculty of Life Sciences, Kumamoto University, 5-1 Oe-honmachi, Chuo-ku, Kumamoto, 862-0973 Japan; 4 AMED-CREST, Japan Agency for Medical Research and Development, 1-7-1 Otemachi, Chiyoda, Tokyo, 100-0004 Japan

## Abstract

Antibiotic-caused changes in intestinal flora (dysbiosis) can have various effects on the host. Secondary bile acids produced by intestinal bacteria are ligands for specific nuclear receptors, which regulate glucose, lipid, and drug metabolism in the liver. The present study aimed to clarify the effect of changes in secondary bile acids caused by antibiotic-induced dysbiosis on the host physiology, especially glucose, lipid, and drug metabolism. After oral administration of non-absorbable antibiotics for 5 days, decreased amounts of secondary bile acid-producing bacteria in faeces and a reduction in secondary bile acid [lithocholic acid (LCA) and deoxycholic acid (DCA)] levels in the liver were observed. Serum glucose and triglyceride levels were also decreased, and these decreases were reversed by LCA and DCA supplementation. Quantitative proteomics demonstrated that the expression levels of proteins involved in glycogen metabolism, cholesterol, bile acid biosynthesis, and drug metabolism (Cyp2b10, Cyp3a25, and Cyp51a1) were altered in the liver in dysbiosis, and these changes were reversed by LCA and DCA supplementation. These results suggested that secondary bile acid-producing bacteria contribute to the homeostasis of glucose and triglyceride levels and drug metabolism in the host, and have potential as therapeutic targets for treating metabolic disease.

## Introduction

Antibiotics are widely used for the treatment and prevention of bacterial infections; however, recent studies indicate that antibiotics can have multiple effects on the host physiology, together with changes in the intestinal flora (dysbiosis)^1,2^. It has been reported that use of antibiotics may increase the risk of developing type-2 diabetes^3^. In addition, hypoglycaemia is known to be an infrequent serious adverse effect of the short-term use of antibiotics^4,5^. Although antibiotics are often prescribed for children, it has been suggested that antibiotic use in infancy or childhood is linked to later weight gain^6–9^. Furthermore, in our previous study, it was revealed that the protein-expression levels of metabolizing enzymes, including cytochrome P450 (cyp) and transporters, were changed in the liver of short-term (5 days) antibiotic-treated mice^10^. Some of the members of the cyp and transporter families are involved in hepatic lipid metabolism and glucose transport^11,12^. Therefore, it is possible that antibiotic-induced dysbiosis affects glucose and lipid homeostasis in the liver.

Bile acids are known to be important for stimulating the absorption of triglycerides (TGs), cholesterol and lipid-soluble vitamins from the intestines, and can also regulate physiology by activation of specific nuclear receptors in the liver^13^. Bile acids are derived from cholesterol in the liver, and released into the duodenum of the small intestine. Then, part of the total amount of the bile acids is metabolized to more hydrophobic and toxic bile acids by intestinal bacteria, such as *Clostridium* cluster XI and XIVa, including *C*. *scindens* and *C*. *sordellii* and *Bacteroides*, including *B*. *fragilis*^14–17^. The principal human primary bile acids, chenodeoxycholic acid (CDCA) and cholic acid (CA) are converted to the secondary bile acids, lithocholic acid (LCA) and deoxycholic acid (DCA), respectively, by bacterial metabolism. These secondary bile acids are absorbed from the colon and returned to the liver via the portal vein.

It has been suggested that nuclear receptors such as the farnesoid X receptor (FXR) are involved in glucose and lipid metabolism and in controlling the expression of metabolic enzymes in the liver. Vitamin D receptor (VDR)-null mice have decreased TG and cholesterol levels in the plasma compared with wild-type mice^18^. Pregnenolone carbonitrile, a murine pregnane X receptor (PXR) agonist, increased hepatic TG and decreased serum glucose levels in wild-type mice, but not in PXR-knockout mice^19,20^. In addition, CYP3A4/3a11 expression is positively regulated by the FXR, the PXR, the constitutive androstane receptor (CAR), and the VDR^21–24^. CYP2B6/2b10 expression is positively regulated by the PXR and CAR; and Cyp51a1 expression is negatively regulated by the FXR^23,25,26^. As bile acids have been reported to be ligands for FXR, PXR, CAR and VDR^16^, it has been inferred that changes in secondary bile acids caused by dysbiosis have an influence on the host physiology, such as glucose, lipid, and drug metabolism, via these nuclear receptors. Although the influence of primary bile acids also needs to be considered, the present study focuses on secondary bile acids, such as LCA and DCA, to elucidate the effect of metabolites produced directly by intestinal bacteria.

The purpose of the present study was to clarify the effect of changes in secondary bile acids caused by antibiotic-induced dysbiosis on the host physiology, especially glucose, lipid and drug metabolism. Antibiotics were administered to mice, with, or without, secondary bile acid supplementation (LCA and DCA), for 5 days and the hepatic expression levels of proteins relating to glucose, lipid and drug metabolism were determined by quantitative proteomic analysis.

## Results

### Reduction of secondary bile acid-producing bacteria caused by antibiotic treatment

Vancomycin (VCM) and polymyxin B (PLB), which are non-absorbable and mainly active against Gram–positive and Gram–negative bacteria, respectively^27–30^, were selected as antibiotics for oral administration to eliminate a broad spectrum of bacteria. VCM and PLB were orally administered for 5 days. To evaluate the function of secondary bile acids under dysbiosis in the present study, we prepared antibiotic-administered mice (AB mice), according to procedures reported in our previous study^10^, and newly prepared antibiotic and secondary bile acid co-administered mice (AB + SB mice), as well as vehicle administered control mice (vehicle control mice; no administration of antibiotics or secondary bile acids). LCA and DCA, the principal human secondary bile acids^17^, were selected as secondary bile acids, and were administered orally at two doses (low and high dose) with antibiotics [AB + SB (low) and AB + SB (high) mice].

**Figure 1:**
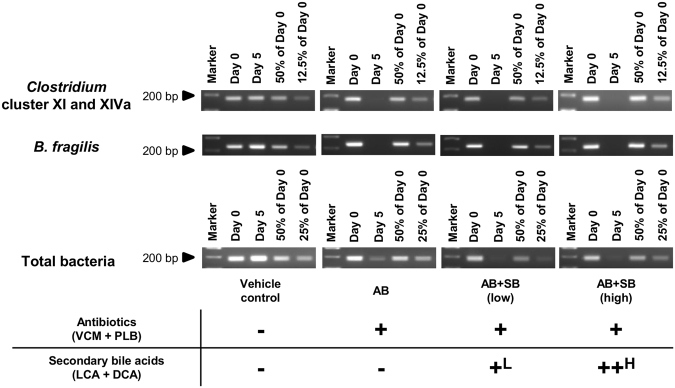
Decrease in the bacterial content of faeces after administration of antibiotics with, or without, secondary bile acid supplementation. The total bacteria, *Clostridium* cluster XI and XIVa, and *B*. *fragilis* content in faeces collected from mice administered antibiotics (VCM + PLB) with, or without, secondary bile acid (LCA + DCA) supplementation for 5 days were analysed by targeted PCR assay of the 16 S rRNA gene from each bacteria (n = 5, the PCR bands from a representative individual in each group are shown). The faeces were collected pre-administration (Day 0) and at 5 days after administration (Day 5), and the templates extracted from a fixed amount of faeces (200 mg) were used for the PCR reaction. For Day 0 faeces, the PCR products amplified from diluted DNA samples [diluted to 50% and 25% (for total bacteria) and 12.5% (for *Clostridium* cluster XI and XIVa and *B*. *fragilis*)], were also analysed as quantitative references. ^L^0.003% LCA and 0.05% DCA, and ^H^0.03% LCA and 0.1% DCA, were mixed with the mouse feed. Full-length gels are presented in Supplementary Figure S2.

A reduction in intestinal bacteria after antibiotic administration was confirmed by PCR assay of the bacterial 16 S ribosomal RNA gene in faeces. In addition to total bacteria, the amount of *Clostridium* cluster XI and XIVa (Gram-positive) and *B*. *fragilis* (Gram-negative), a representative bacteria of the *Bacteroides* species, were evaluated as bacteria producing secondary bile acids^14–16,31^. In the antibiotic-treated group (AB, AB + SB (low), and AB + SB (high) mice; n = 5), the amounts of *Clostridium* cluster XI and XIVa, *B*. *fragilis*, and total bacteria after treatment for 5 days were decreased to less than 12.5%, 12.5%, and 50% of pre-administration amounts, respectively (Fig. 1 and Supplementary Figure S1). There was no change in the amount of these bacteria in vehicle control mice after 5 days. The vehicle control, AB, AB + SB (low), and AB + SB (high) mice were used in the following experiments.

### Reduction and recovery of secondary bile acid levels in the liver by antibiotic treatment and secondary bile acid supplementation

The concentrations of secondary bile acids in the liver were determined, to confirm the reduction of secondary bile acid levels in the liver under antibiotic-induced dysbiosis, and recovery of hepatic secondary bile acid levels by supplementation. Taurolithocholic acid (TLCA) and taurodeoxycholic acid (TDCA) were analysed because most LCA and DCA molecules exist as taurine conjugates in the mouse liver^32^. The concentrations of taurochenodeoxycholic acid (TCDCA) and taurocholic acid (TCA), which are taurine conjugates of primary bile acids of LCA and DCA, respectively, and the concentration of tauro-β-muricholic acid (TβMCA), which is a taurine conjugate of primary bile acid present abundantly in mouse liver^32^, were determined together with TLCA and TDCA.

**Figure 2:**
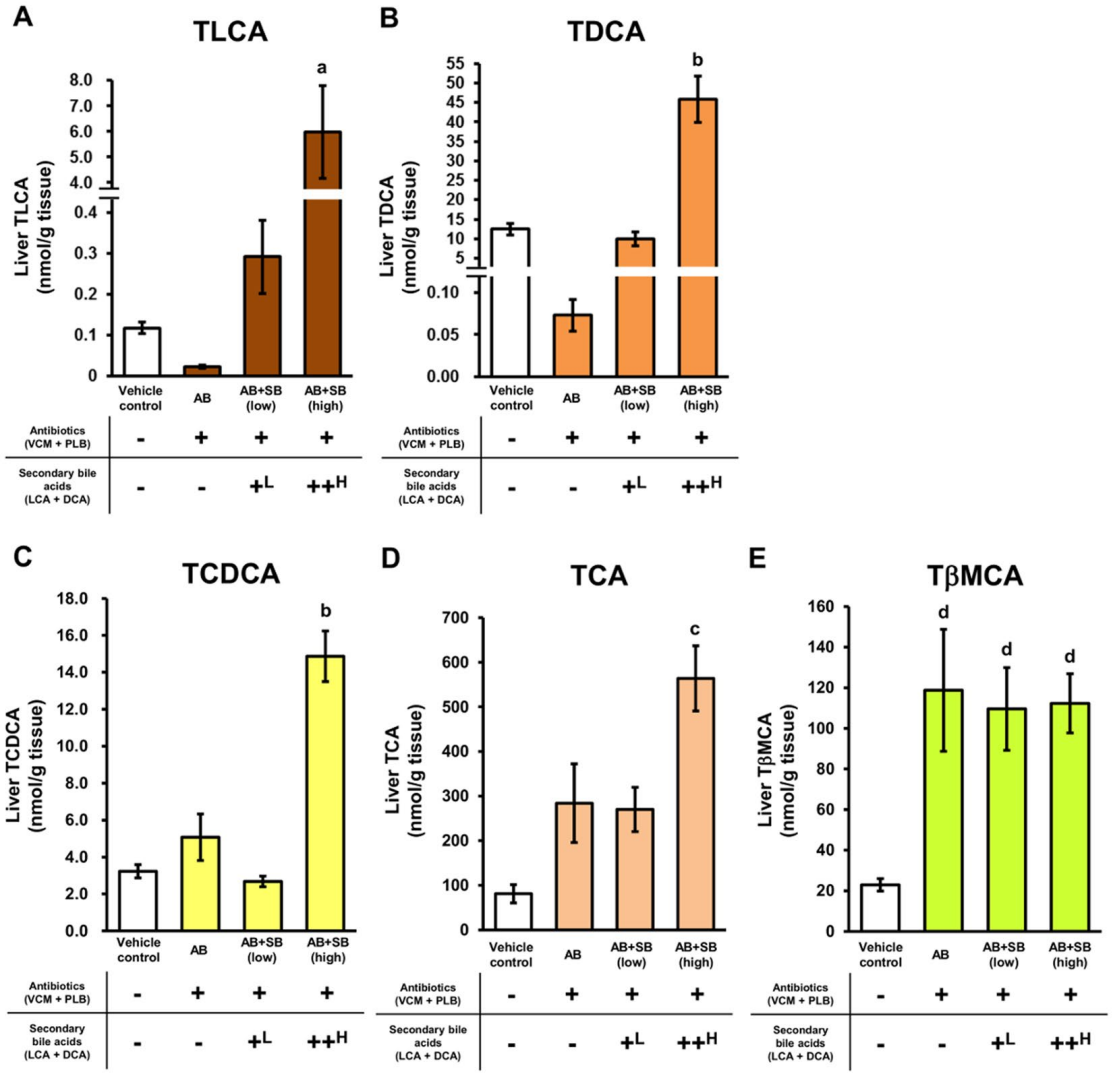
Bile acid levels in the liver after administration of antibiotics with, or without, secondary bile acid supplementation. TLCA (**A**), TDCA (**B**), TCDCA (**C**), TCA (**D**), and TβMCA (**E**) levels are presented as the mean ± SEM (n = 5). ^a^Significantly different from vehicle control, AB, and AB + SB (low) mice (P < 0.01). ^b^Significantly different from vehicle control, AB, and AB + SB (low) mice (P < 0.001). ^c^Significantly different from vehicle control (P < 0.001), AB, and AB + SB (low) mice (P < 0.05). ^d^Significantly different from vehicle control mice (P < 0.05). ^L^0.003% LCA and 0.05% DCA, and ^H^0.03% LCA and 0.1% DCA, were mixed with the mouse feed.

The TLCA and TDCA levels in the livers of AB mice were decreased to 19.8% and 0.586% compared with those in vehicle control mice, respectively (Fig. 2A,B). These decreases in the TLCA and TDCA levels were recovered to 248% and 79.5% of those in vehicle control mice, respectively, in AB + SB (low) mice. In AB + SB (high) mice, the TLCA and TDCA levels were increased by 51.0- and 3.68-fold compared with vehicle control mice, respectively. In contrast, the TCDCA, TCA and TβMCA levels of AB mice were increased by 1.57-, 3.51-, and 5.18-fold compared with vehicle control mice, respectively (Fig. 2C–E). The TCDCA and TCA levels in AB + SB (high) mice were increased by 4.61- and 6.96-fold compared with vehicle control mice, respectively.

### Effects of dysbiosis caused by antibiotics and secondary bile acid supplementation on host physiology

**Figure 3:**
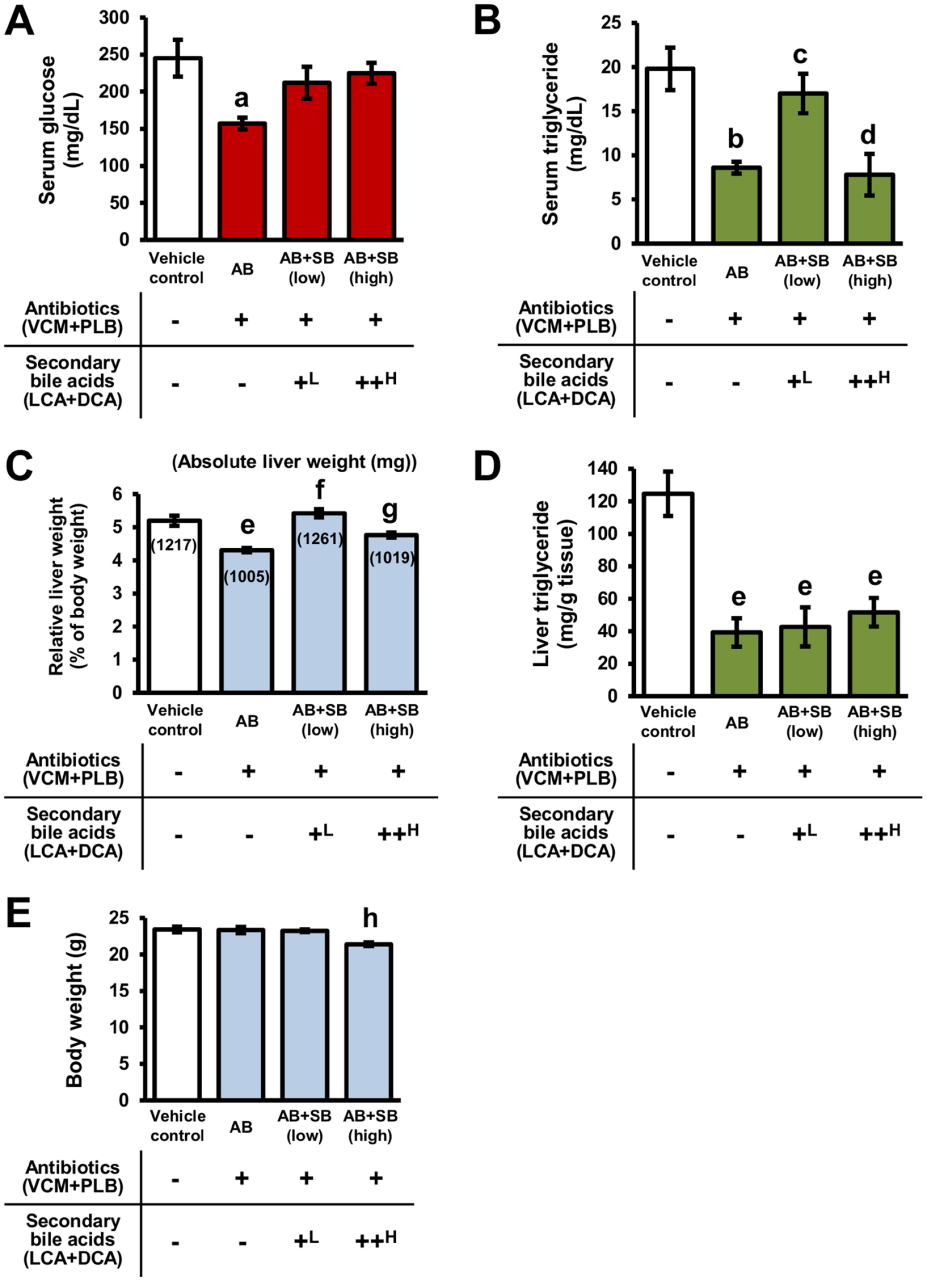
Effects of dysbiosis caused by antibiotics, and secondary bile acid supplementation, on serum glucose levels (**A**), serum TG levels (**B**), liver weight (**C**), liver TG levels (**D**), and body weight (**E**). Data are presented as the mean ± SEM (n = 5). ^a^Significantly different from vehicle control mice (P < 0.05). ^b^Significantly different from vehicle control mice (P < 0.01). ^c^Significantly different from AB mice (P < 0.05). ^d^Significantly different from vehicle control (P < 0.01) and AB + SB (low) mice (P < 0.05). ^e^Significantly different from vehicle control mice (P < 0.001). ^f^Significantly different from AB mice (P < 0.001). ^g^Significantly different from AB (P < 0.05) and AB + SB (low) mice (P < 0.01). ^h^Significantly different from vehicle control, AB, and AB + SB (low) mice (P < 0.01). ^L^0.003% LCA and 0.05% DCA, and ^H^0.03% LCA and 0.1% DCA, were mixed with the mouse feed.

The effects of hepatic secondary bile acid levels in dysbiosis on the host physiology were examined. The results of biochemical blood tests, as well as liver weights, liver TG levels, and body weights of the mice administered antibiotics with, or without, secondary bile acid supplementation are shown in Fig. 3 and Table 1. Information regarding food intake is also shown in Supplementary Figure S3. On day 0 and 5 days after administration, there was a difference in food intake of 2.07 times or less in mice treated with secondary bile acid compared with the vehicle control and antibiotic-treated mice, but there was no significant difference between the groups 2 to 4 days after administration.

**Table 1:** Biochemical blood tests in mice administered antibiotics with, or without, secondary bile acid supplementation^a^.

Antibiotics (VCM + PLB)	−	+	+	+
Secondary bile acids (LCA + DCA)	−	−	+^L^	++^H^
	Vehicle control	AB	AB + SB (low)	AB + SB (high)
**LDH** (IU/L)	850 ± 315	951 ± 211	840 ± 226	942 ± 418
**AST** (IU/L)	64.4 ± 13.1	66.2 ± 11.6	85.8 ± 17.6	97.4 ± 10.9
**ALT** (IU/L)	37.6 ± 7.7	31.6 ± 9.9	57.8 ± 12.9	63.6 ± 5.1
**ALP** (IU/L)	347 ± 17	301 ± 12	350 ± 27	294 ± 14
**γ-GTP** (IU/L)	0 ± 0	0 ± 0	0 ± 0	0 ± 0
**TP** (g/dL)	4.11 ± 0.03	4.12 ± 0.05	4.36 ± 0.07	4.57 ± 0.16^b^
**ALB** (g/dL)	0.656 ± 0.031	0.708 ± 0.007	0.712 ± 0.012	0.824 ± 0.053^c^
**T-BIL** (mg/dL)	0.026 ± 0.007	0.018 ± 0.011	0.006 ± 0.004	0.048 ± 0.025
**CK** (IU/L)	203 ± 45	303 ± 84	367 ± 132	250 ± 52
**AMY** (IU/L)	1659 ± 114	1703 ± 81	1752 ± 74	1826 ± 130
**T-CHO** (mg/dL)	59.0 ± 1.8	56.6 ± 1.4	68.4 ± 2.8	71.2 ± 5.2^d^
**HDL-C** (mg/dL)	39.7 ± 1.2	37.9 ± 1.4	31.0 ± 1.2	30.3 ± 3.9^e^
**LDL-C** (mg/dL)	6.8 ± 0.4	7.0 ± 0	11.2 ± 1.1^b^	10.6 ± 1.3^b^
**UN** (mg/dL)	20.2 ± 1.3	22.3 ± 1.5	28.2 ± 2.8^e^	19.6 ± 1.5 ^f^
**UA** (mg/dL)	2.34 ± 0.07	2.34 ± 0.16	3.02 ± 0.24	3.42 ± 0.27 ^g^
**CRE** (mg/dL)	0.122 ± 0.005	0.160 ± 0.017	0.174 ± 0.017^e^	0.134 ± 0.005
**Ca** (mg/dL)	8.50 ± 0.03	7.72 ± 0.41	8.78 ± 0.12^d^	9.18 ± 0.19 ^h^
**IP** (mg/dL)	9.62 ± 0.49	8.38 ± 0.29	8.90 ± 0.42	8.24 ± 0.36
**Na** (mM)	146 ± 1	146 ± 1	148 ± 1	149 ± 2
**K** (mM)	9.8 ± 0.6	9.2 ± 0.2	10.3 ± 0.2	10.1 ± 1.0
**Cl** (mM)	105.1 ± 0.5	104.6 ± 0.4	106.6 ± 1.1	104.4 ± 0.7

^a^Biochemical blood tests of mice administered antibiotics (VCM + PLB) with, or without, secondary bile acid supplementation (LCA + DCA) for 5 days are presented as the mean ± SEM (n = 5). ^b^Significantly different from vehicle control and AB mice (P < 0.05). ^c^Significantly different from vehicle control mice (P < 0.01). ^d^Significantly different from AB mice (P < 0.05). ^e^Significantly different from vehicle control mice (P < 0.05). ^f^Significantly different from AB + SB (low) mice (P < 0.05). ^g^Significantly different from vehicle control and AB mice (P < 0.01). ^h^Significantly different from AB mice (P < 0.01). ^L^0.003% LCA and 0.05% DCA, and ^H^0.03% LCA and 0.1% DCA, were mixed with the mouse feed.

LDH, lactate dehydrogenase; AST, aspartate aminotransferase; ALT, alanine aminotransferase; ALP, alkaline phosphatase; γ-GTP, γ-glutamyl transpeptidase; TP, total protein; ALB, albumin; T-BIL, total bilirubin; CK, creatine kinase; AMY, α-amylase; T-CHO, total cholesterol; HDL-C, high density lipoprotein cholesterol; LDL-C, low density lipoprotein cholesterol; UN, urea nitrogen; UA, uric acid; CRE, creatinine; Ca, calcium; IP, inorganic phosphorus; Na, sodium; K, potassium; Cl, chloride.

As shown in Fig. 3A,B, serum glucose and TG levels in AB mice were decreased to 64.1% and 43.4% of vehicle control mice, respectively. Other biochemical parameters in the serum of AB mice were not significantly changed compared with vehicle control mice (Table 1). Liver weight and hepatic TG levels in AB mice were also decreased to 82.9% and 31.4% of vehicle control mice, respectively (Fig. 3C,D). The decreases in serum glucose levels and liver weight were recovered in AB + SB (low) and AB + SB (high) mice (Fig. 3A and C). The decrease in serum TG levels was recovered in only AB + SB (low) mice, and hepatic TG levels were not recovered by co-administration with secondary bile acids in any of the mice (Fig. 3B and D). Body weight in mice was not significantly affected in AB and AB + SB (low) mice; the body weight of AB + SB (high) mice was decreased to 91.5% of vehicle control mice (Fig. 3E).

### Effects of dysbiosis caused by antibiotics, and secondary bile acid supplementation, on the hepatic expression of proteins in cholesterol and bile acid biosynthetic pathways

To explore the mechanisms for the alterations in host physiology caused by exposure to hepatic secondary bile acids in dysbiosis, the protein expression levels in the livers of mice administered antibiotics with, or without, secondary bile acid supplementation were quantified comprehensively. Mouse liver was fractionated to cytosol, crude membrane (containing all membrane fractions except for the nuclear membrane) and plasma membrane fractions, and each fraction was analysed by focused quantitative proteomics. Focused quantitative proteomics of the subcellular fractions of biological samples improved the detection sensitivity by concentrating the proteins in the fractions, making it possible to analyse changes in the subcellular localization of the proteins. The purity of the fractionated fractions was assessed by the intensities of the marker proteins (Supplementary Figure S4). Among the fractionated fractions, hypoxanthine-guanine phosphoribosyltransferase (Hprt1, cytosol fraction marker), cytochrome c oxidase subunit 4 (Cox4, crude membrane fraction marker), and Na^+^/K^+^-ATPase (plasma membrane fraction marker) were detected at the highest intensity in the cytosol, crude membrane, and plasma membrane fractions, respectively. In the liver of model mice, 1147, 1246, 1320 and 1929 proteins were identified in the cytosol, crude membrane, plasma membrane and total fractions, respectively. We focused on the proteins that had significantly increased or decreased expression in AB mice compared with vehicle control mice, and had significantly decreased or increased expression, respectively, in AB + SB (low) mice compared with AB mice (p < 0.05). There were 36, 65, 71, and 161 such proteins identified in the cytosol, crude membrane, plasma membrane, and total fractions, respectively, and these proteins are listed in Supplementary Tables S1 and S2.

Functional annotation clustering and enrichment analysis was performed for the 161 identified proteins that exhibited significant expressional changes. The results showed that proteins involved in sterol, cholesterol and steroid biosynthesis were enriched in the identified proteins. More than half of the enriched proteins involved in these biosyntheses were increased in AB mice compared with vehicle control mice and were decreased in AB + SB (low) mice compared with AB mice (Table 2).

**Table 2:** Functional annotation clustering and enrichment analysis of proteins with expression levels that were significantly changed in the liver by antibiotic administration that were recoverable by secondary bile acid supplementation^a^.

Term	P-value	FDR	Protein count
oxidation reduction↑	6.36E-15	9.82E-12	34
sterol biosynthetic process↑	7.54E-12	1.18E-08	10
cholesterol biosynthetic process↑	2.95E-11	4.61E-08	9
steroid biosynthetic process↑	1.60E-09	2.49E-06	11
translation↑	2.01E-09	3.14E-06	19

^a^The analysis was performed using DAVID for proteins with expression levels that were significantly increased or decreased in AB mice compared with vehicle control mice and were significantly decreased or increased, respectively, in AB + SB (low) mice compared with AB mice.

↑: More than half of the proteins were significantly increased in AB mice and were significantly decreased in AB + SB (low) mice.

**Figure 4:**
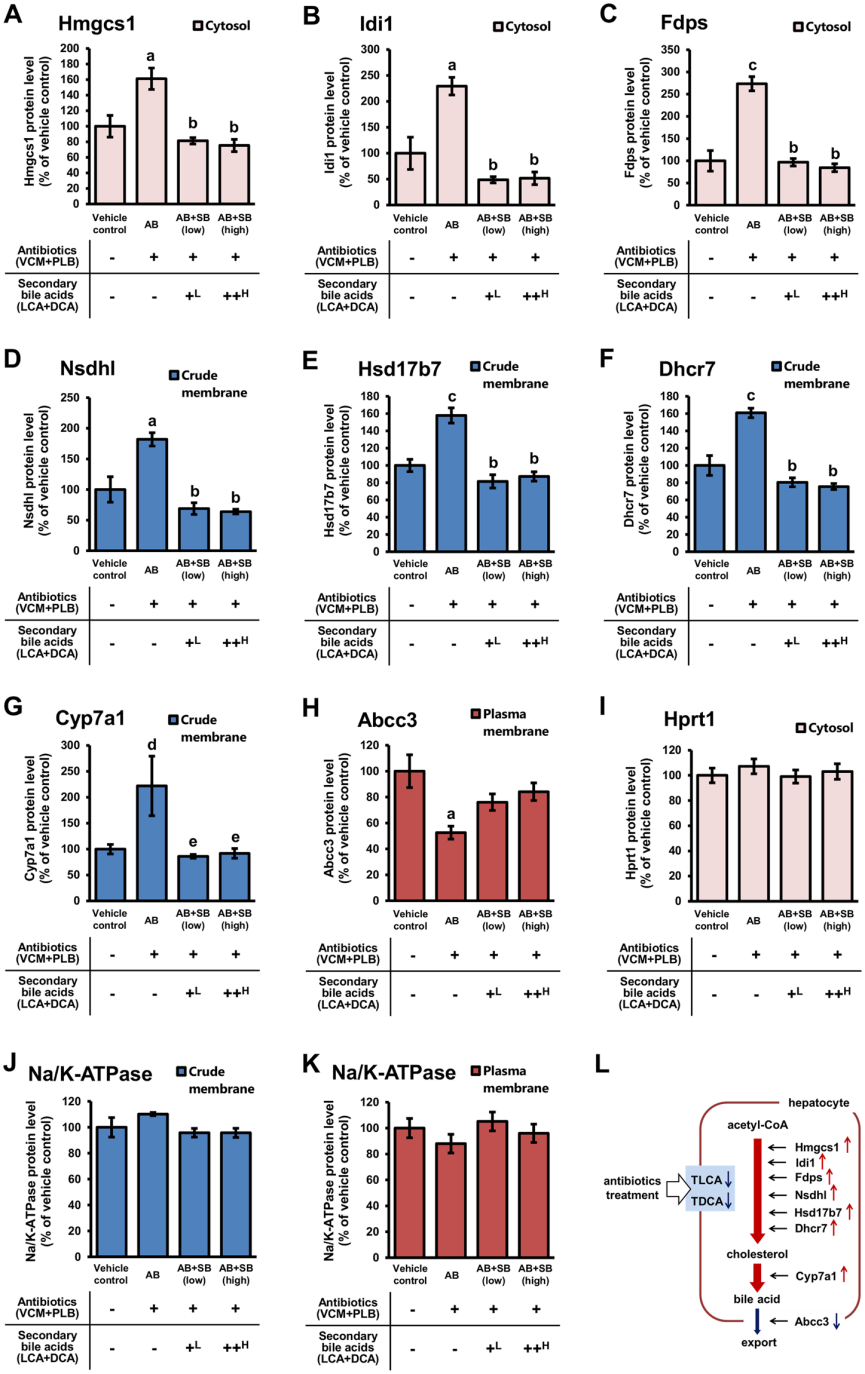
Expression levels of proteins involved in the biosynthesis and excretion pathways of cholesterol and bile acid in the liver. The expression levels of proteins involved in biosynthesis and excretion pathway of cholesterol and bile acid (**A**–**H**), and housekeeping proteins (**I**–**K**), in each hepatocellular fraction prepared from mice administered antibiotics (VCM + PLB) with, or without, secondary bile acid (LCA + DCA) supplementation were determined by focused quantitative proteomics. Relative protein expression levels compared with those in vehicle control mice are presented as the mean ± SEM (n = 5). ^a^Significantly different from vehicle control mice (P < 0.01). ^b^Significantly different from AB mice (P < 0.001). ^c^Significantly different from vehicle control mice (P < 0.001). ^d^Significantly different from vehicle control mice (P < 0.05). ^e^Significantly different from AB mice (P < 0.05). A scheme showing altered protein levels in the biosynthesis and excretion pathways of cholesterol and bile acid in the hepatocytes of AB mice (L). ^L^0.003% LCA and 0.05% DCA, and ^H^0.03% LCA and 0.1% DCA, were mixed with the mouse feed.

As shown in Fig. 4, the expression levels of hepatic proteins involved in the cholesterol biosynthetic pathway, including hydroxymethylglutaryl-CoA synthase, cytoplasmic (Hmgcs1), isopentenyl-diphosphate Delta-isomerase 1 (Idi1), farnesyl pyrophosphate synthase (Fdps), sterol-4-alpha-carboxylate 3-dehydrogenase, decarboxylating (Nsdhl), 3-keto-steroid reductase (Hsd17b7) and 7-dehydrocholesterol reductase (Dhcr7)^11^, were increased in AB mice compared with vehicle control mice, and this increase was reversed by secondary bile acid supplementation (Fig. 4A–F). In addition, the protein expression of Cyp7a1, which is involved in a rate-limiting step in the biosynthesis of bile acids from cholesterol, was also altered in the AB mice (Fig. 4G). Canalicular multispecific organic anion transporter 2 (Abcc3, also known as Mrp3), which is a basolateral efflux transporter that exports conjugated bile acid^33^, was decreased in AB mice compared with vehicle control mice, and this decrease was reversed by secondary bile acid supplementation (Fig. 4H). These results suggested that the protein expression of the molecules shown in Fig. 4A–H was regulated by secondary bile acids directly or indirectly. Furthermore, as shown in Fig. 4L, the changes in protein profile in the pathways relating to cholesterol and bile acid indicate the possibility that the production of cholesterol and bile acid in hepatocytes was induced by dysbiosis caused by antibiotic treatment.

The expression levels of Hprt1, which is housekeeping protein^34^, were not significantly different in AB, AB + SB (low) and AB + SB (high) mice compared with vehicle control mice (99.1%–107% of vehicle control mice) in the cytosol fraction (Fig. 4I). The expression levels of Na^+^/K^+^-ATPase, a housekeeping protein^35^, in the crude membrane and plasma membrane fractions were not significantly different (95.7–110% and 88.0–105% of vehicle control mice, respectively) (Fig. 4J,K). These results suggest that the preparation of each hepatocellular fraction was not significantly different among the groups.

### Effects of dysbiosis caused by antibiotics and secondary bile acid supplementation on hepatic protein expression levels in the glycogen metabolism pathway

**Figure 5:**
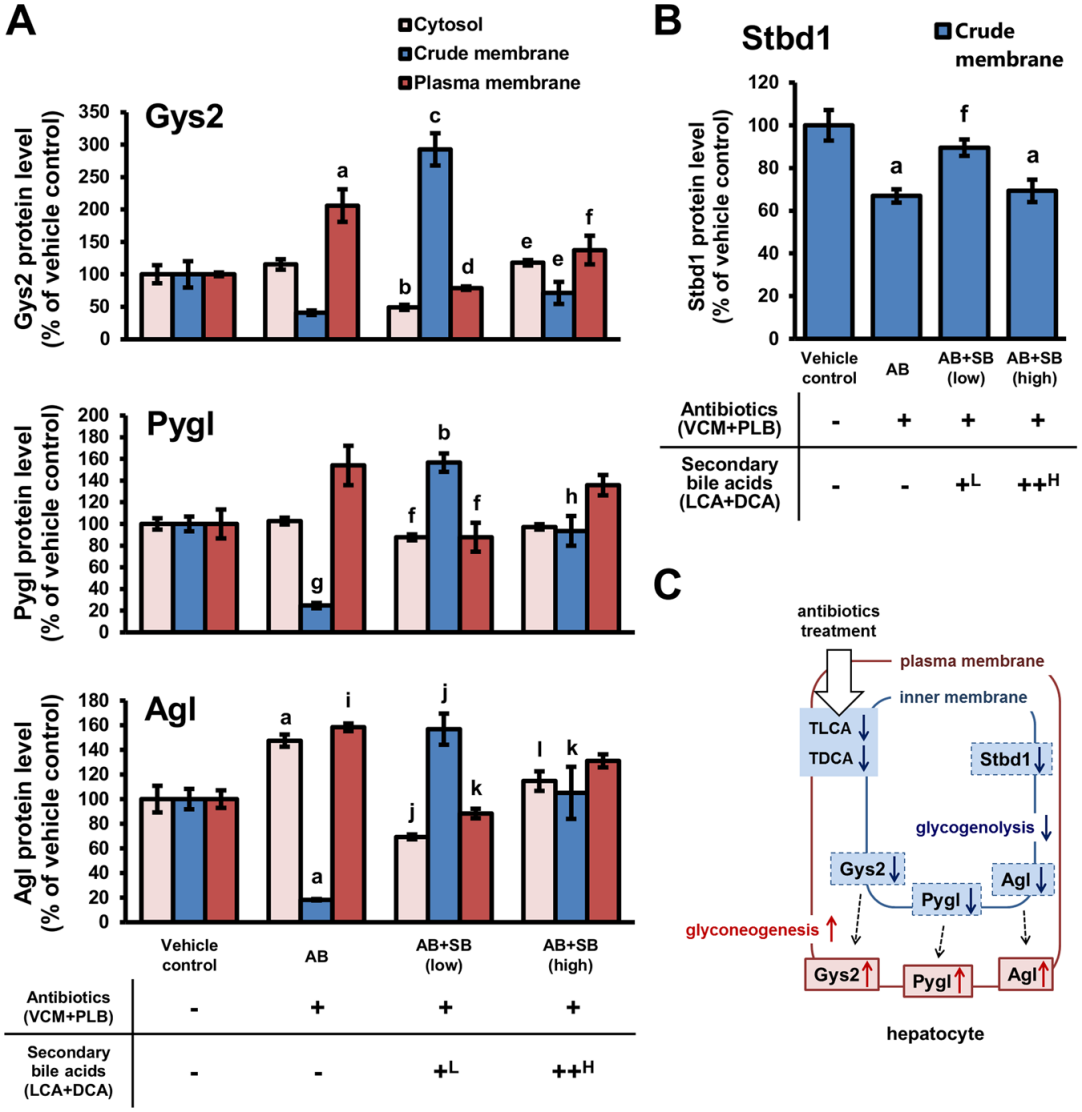
Expression levels of proteins involved in the glycogen metabolic pathway in the liver. The protein expression levels of Gys2, Pygl, Agl (**A**) and Stbd1 (**B**) in liver fractions prepared from mice administered antibiotics (VCM + PLB) with, or without, secondary bile acid supplementation (LCA + DCA) were analysed by focused quantitative proteomics. Relative protein expression levels compared with those in vehicle control mice are presented as the mean ± SEM (n = 5). ^a^Significantly different from vehicle control mice (P < 0.01). ^b^Significantly different from vehicle control (P < 0.01) and AB mice (P < 0.001). ^c^Significantly different from vehicle control and AB mice (P < 0.001). ^d^Significantly different from AB mice (P < 0.001). ^e^Significantly different from AB + SB (low) mice (P < 0.001). ^f^Significantly different from AB mice (P < 0.05). ^g^Significantly different from vehicle control mice (P < 0.001). ^h^Significantly different from AB and AB + SB (low) mice (P < 0.001). ^i^Significantly different from vehicle control mice (P < 0.05). ^j^Significantly different from vehicle control (P < 0.05) and AB mice (P < 0.001). ^k^Significantly different from AB mice (P < 0.01). ^l^Significantly different from AB (P < 0.05) and AB + SB (low) mice (P < 0.01). A scheme showing changes in the protein expression levels in AB mice (**C**). ^L^0.003% LCA and 0.05% DCA, and ^H^0.03% LCA and 0.1% DCA, were mixed with the mouse feed.

As shown in Fig. 3A, serum glucose levels were decreased in dysbiosis and the level was able to be recovered by secondary bile acid supplementation, suggesting that reduction of secondary bile acid in dysbiosis affects glucose-related homeostasis. Focused quantitative proteomics revealed that levels of proteins related to the glycogen biosynthesis pathway were changed by antibiotic-induced dysbiosis and secondary bile acid supplementation. Glycogen [starch] synthase, liver (Gys2) is involved in the pathway for glycogen biosynthesis, and its translocation to the cell periphery, at the inner face of the plasma membrane, initiates glycogen synthesis in hepatocytes^36,37^. As shown in Fig. 5A, Gys2 expression in the plasma membrane fraction of AB mice was increased by 2.06-fold, while expression in the crude membrane fraction was decreased to 40.9%, compared with control, suggesting the possibility that Gys2 was translocated to the cell periphery and induced the glycogen synthesis in dysbiosis caused by antibiotic treatment. In AB + SB (low) mice, Gys2 levels in the plasma membrane fraction were recovered to control levels, while expression in the crude membrane fraction was increased by 2.93-fold compared with vehicle control mice, suggesting Gys2 was accumulated in the intracellular components.

In glycogenolysis, the main enzymes involved are glycogen phosphorylase, liver form (Pygl) and glycogen debranching enzyme (Agl)^38^. The generated glycogen is transferred to the intracellular region, and then is degraded by Pygl and Agl in the cytoplasm. The change in expression levels of Pygl and Agl showed a similar tendency to that of Gys2 (Fig. 5A). In AB mice, the expression levels of Pygl and Agl were increased in the plasma membrane fraction by 1.54- and 1.58-fold, respectively, while expression levels in the crude membrane fraction were decreased to 24.7% and 18.3%, respectively, compared with vehicle control mice. These results suggest the possibility that Pygl and Agl were translocated to the cell periphery and hence, glycogenolysis was expected to be suppressed in the intracellular region of the hepatocytes in AB mice. In AB + SB (low) mice, the expression levels of Pygl and Agl in the plasma membrane fraction were recovered to control levels, and expression in the crude membrane fraction was increased by 1.57-fold compared with vehicle control mice, suggesting accumulation of Pygl and Agl in the intracellular components.

Part of the total amount of glycogen is degraded in the lysosomes and the starch-binding domain-containing protein 1 (Stbd1) plays a role in glycogen transport to lysosomes in the liver^39^. In the present study, the levels of Stbd1 were decreased to 60.9% of vehicle control mice in the crude membrane fraction of AB mice (Fig. 5B). This decrease in Stbd1 levels was recovered in AB + SB (low) mice to 89.6% of vehicle control mice.

### Effects of secondary bile acid supplementation on the protein expression levels of cytochrome P450 enzymes

Previously we have demonstrated that the protein expression levels of Cyp2b10, Cyp3a11 and Cyp3a25 were decreased, and Cyp51a1 was increased, in the liver of antibiotic-treated mice^10^. CYP2B6 and CYP3A4 are the human homologues of Cyp2b10 and Cyp3a11, respectively, and over half of therapeutic drugs are metabolized by either the CYP2B6 or CYP3A subfamily^40,41^. Cyp51a1 is a sterol 14α-demethylase involved in biosynthetic pathway of cholesterol^42^. To clarify the regulation of Cyp enzymes in AB mice by secondary bile acids, the protein expression levels of Cyp enzymes in the crude membrane fraction of the liver were measured by targeted quantitative proteomics using stable isotope-labelled internal standard peptides.

**Figure 6:**
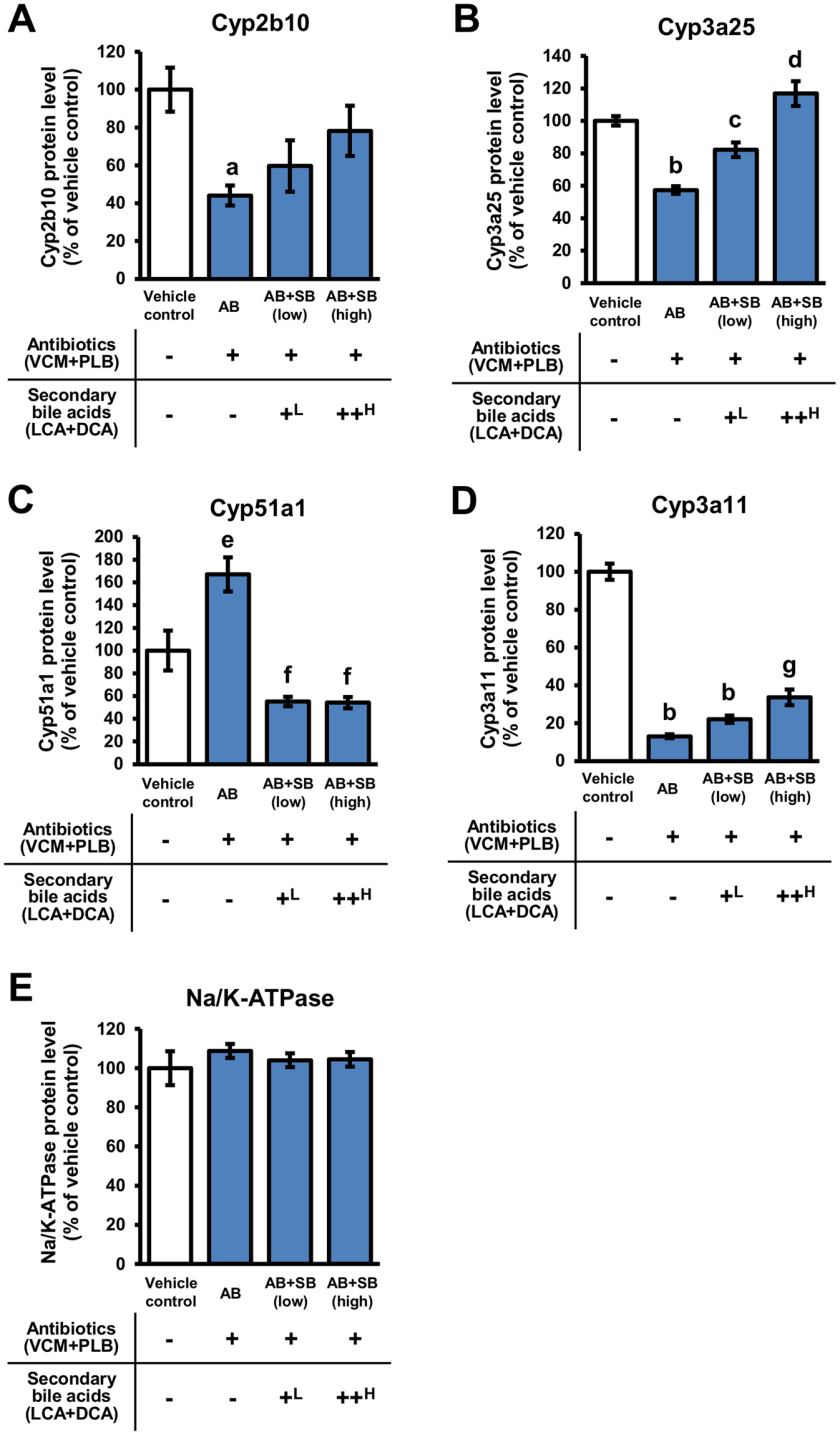
The protein expression levels of Cyp enzymes in the liver. The protein expression levels of Cyp2b10, Cyp3a25, Cyp51a1, Cyp3a11, and Na/K-ATPase in liver crude membrane fractions prepared from mice administered antibiotics (VCM + PLB) with, or without, secondary bile acid supplementation (LCA + DCA) were analysed by targeted proteomics. Relative protein expression levels compared with those in vehicle control mice are presented as the mean ± SEM (n = 5). ^a^Significantly different from vehicle control mice (P < 0.05). ^b^Significantly different from vehicle control mice (P < 0.001). ^c^Significantly different from AB mice (P < 0.05). ^d^Significantly different from AB and AB + SB (low) mice (P < 0.001). ^e^Significantly different from vehicle control mice (P < 0.01). ^f^Significantly different from AB mice (P < 0.001). ^g^Significantly different from vehicle control (P < 0.001) and AB mice (P < 0.01).^L^0.003% LCA and 0.05% DCA, and ^H^0.03% LCA and 0.1% DCA, were mixed with the mouse feed.

Levels of Cyp2b10 in AB mice were decreased to 44.0% of vehicle control mice, and the decrease was recovered to 59.7% and 78.2% of vehicle control mice in AB + SB (low) and AB + SB (high) mice, respectively (Fig. 6A). Cyp3a25 levels in AB mice were also decreased to 57.3% of vehicle control mice, and the decrease was recovered to 82.2% and 117% of vehicle control mice in AB + SB (low) and AB + SB (high) mice, respectively (Fig. 6B). Cyp51a1 levels in AB mice were increased by 1.67-fold compared with vehicle control mice, and levels in AB + SB (low) and AB + SB (high) mice were decreased to 55.2% and 54.2% of vehicle control mice, respectively (Fig. 6C). In contrast, Cyp3a11 levels in AB mice were decreased to 13.1% of vehicle control mice, and the decrease was slightly recovered to 22.1% and 33.7% of vehicle control mice in AB + SB (low) and AB + SB (high) mice, respectively (Fig. 6D). The expression levels of Na^+^/K^+^-ATPase in AB, AB + SB (low), and AB + SB (high) mice were not significantly different compared with vehicle control mice (104%–109% of vehicle control mice) (Fig. 6E).

## Discussion

The present study revealed that serum glucose levels, serum and liver TG levels, and liver weights in conventional mice were decreased by short-term antibiotic administration (5 days) (Fig. 3A–D). The decreases in serum glucose and TG levels, and liver weights were able to be recovered with low dose secondary bile acid supplementation, which recovered the hepatic levels of TLCA and TDCA to control levels, suggesting that secondary bile acids generated by intestinal bacteria contribute to these changes. It has been reported that antibiotic administration (norfloxacin and ampicillin for 2 weeks) to disease model mice (*ob/ob* mice) caused decreases in blood glucose and liver TG levels^43^. In this study, both blood glucose and liver TG levels were decreased, even in conventional normal mice, by antibiotic administration for a shorter term (5 days). Thus, short-term treatment with antibiotics has the potential to decrease blood glucose and liver TG levels in normal humans.

Hypoglycaemia is known to be a serious adverse effect of fluoroquinolones, which are broad-spectrum antibiotics, and typically occurs within the short-term (first 3 days of administration)^4,5^. An increase in insulin secretion by inhibition of the ATP-sensitive potassium channels of pancreatic islet cells by fluoroquinolones has been reported as a possible mechanism for hypoglycaemia. The present study provides evidence for an additional mechanism involving induction of glycogen biosynthesis in the liver by a reduction in secondary bile acids caused by dysbiosis. As shown in Fig. 5A, the possibility of translocation of Gys2, Pygl and Agl proteins in hepatocytes was suggested to be induced by reductions in hepatic secondary bile acid levels under dysbiosis conditions in AB mice. It has been reported that Gys2 generates glycogen at the cell cortex, and Pygl and Agl degrade glycogen at intracellular sites^36–38^. Therefore, the translocation of Gys2, Pygl, and Agl to the plasma membrane fraction from the crude membrane fraction results in enhancement of glycogen synthesis in the liver and then a decrease in serum glucose levels. It has also been shown in a previous study that administration of antibiotics to *ob/ob* mice for 2 weeks increased glycogen levels in the liver^43^. However, further data, such as immunohistochemistry of Gys2, Pygl, and Agl, are necessary to confirm the regulation of glycogen synthesis by secondary bile acid. In the liver, glycogen degradation is also conducted by transport via Stbd1 to lysosomes in an autophagy-like process^39^. Therefore, the observed decrease in Stbd1 levels in the liver of AB mice also contributes to the suppression of glycogen degradation (Fig. 5B).

Among the bile acid-activated nuclear receptors in liver, it has been reported that PXR and VDR activation increases plasma TG levels^44^. In this study, serum TG levels were decreased by antibiotic administration, with a reduction in hepatic secondary bile acid levels (Fig. 3B). Since LCA has a higher ligand activity against PXR and VDR than other bile acids^21,45^, a decrease in secondary bile acid levels in the liver is likely to reduce PXR and VDR activity, and consequently decrease serum TG levels. The observed decrease in hepatic TG levels was not able to be recovered with secondary bile acid supplementation (Fig. 3D). A previous study has reported that decreases in hepatic TG levels after antibiotic treatment was caused by elevated plasma adiponectin levels from adipose tissue^43^.

The expression levels of proteins involved in the biosynthetic pathway of cholesterol in hepatocytes were altered in AB mice, accompanying the reductions in secondary bile acid (Fig. 4). The expression level of Cyp7a1, which plays a rate-limiting step in the biosynthesis of bile acid from cholesterol, was increased in AB mice and supressed to control levels in AB + SB mice (Fig. 4G). Since the expression of Cyp7a1 is suppressed by activation of FXR^46^, the present results suggest that the bile acid profile became antagonistic to FXR in AB mice and recovered to control levels in AB + SB mice. Similarly, activation of FXR is involved in liver regeneration^47^. As shown in Fig. 3C, the liver weight of AB mice was decreased, and this decrease was recovered by low- and high-dose secondary bile acid supplementation, supporting an FXR-antagonistic bile acid profile in AB mice. It has also been reported that FXR regulates cholesterol levels in the blood by regulating the expression of scavenger receptor B1 (SR-B1) in the liver^44,48^. However, as shown in Table 1, the serum levels of total cholesterol (T-CHO), high density lipoprotein cholesterol (HDL-C), and low density lipoprotein cholesterol (LDL-C) did not alter in AB mice, while in AB + SB mice, the levels of T-CHO and LDL-C tended to be increased and the levels of HDL-C tended to be decreased. Since CAR, PXR, and VDR are also involved in the regulation of blood cholesterol levels^44^, the changes in bile acid profile in AB and AB + SB mice are likely to affect the serum levels of T-CHO, HDL-C, and LDL-C by the involvement of complex regulation via multiple nuclear receptors.

In previous studies, it has been suggested that high-dose administration of secondary bile acids (LCA; 0.03%–0.3% of diet, DCA; 0.1%–0.3% of diet) caused hepatic dysfunction, as shown by an increase in serum alanine aminotransferase (ALT) levels in mice, and periportal inflammatory infiltration has been reported by histological analysis of the liver when DCA (0.5%–1%) was administered to rats^49,50^. Indeed, serum aspartate aminotransferase (AST) and ALT levels tended to be increased depending on the secondary bile acid supplementation dose in the present study (Table 1). Body weight was decreased in AB + SB (high) mice, and the observed decrease in serum TG levels was not recovered in AB mice by high-dose secondary bile acid supplementation (Fig. 3B and E). It was inferred from these results that exposure to hepatic secondary bile acids at physiological levels contributes to host homeostasis, while exposure to hepatic secondary bile acids over physiological levels could be toxic for liver function.

The protein expression levels of Cyp2b10, Cyp3a25, and Cyp51a1 in the liver were influenced by supplementation with secondary bile acids (Fig. 6A–C), suggesting that the changes in protein expression of these molecules were mainly because of the reduction in secondary bile acids caused by dysbiosis in AB mice. In contrast, the reduction in Cyp3a11 levels in AB mice was not reversed by secondary bile acid supplementation (Fig. 6D). This suggests that factors other than secondary bile acids were involved in the suppression of Cyp3a11 expression in AB mice. Previous reports have suggested that polysaccharide A and butyrate, which are produced by intestinal bacteria, protected mice from colitis^51,52^. In another study, antibiotic administration (streptomycin or vancomycin administered orally for 2 days) led to copious inflammatory infiltration of the intestines of mice, in a dose-dependent manner, and caused more severe intestinal pathology with increasing susceptibility to enteric infection^53^. Therefore, dysbiosis was considered to promote inflammation of the intestine. In addition, it has been reported that mRNA and protein expression levels of Cyp3a were decreased in mice with dextran sodium sulfate-induced colitis^54^. From these reports, the decrease in expression levels of Cyp3a11 in AB mice is likely to be attributed to inflammation of the intestine induced by dysbiosis.

In the present study, administration of antibiotics (co-administration of VCM and PLB) reduced TLCA and TDCA levels in the mice livers (Fig. 2A,B). It was expected that the amount of primary bile acids increased due to the decrease in secondary bile acids by antibiotic administration, because the expression of proteins involved in the synthesis of cholesterol and bile acids was increased by the reduction of secondary bile acid by antibiotic administration and suppressed to the control levels by the supplementation of secondary bile acids (Fig. 4). Indeed, in the present study, the hepatic levels of TCDCA, TCA, and TβMCA were increased by administration of antibiotics (Fig. 2C–E). TCDCA and CDCA, the primary bile acid of LCA, have stronger ligand activity to FXR than LCA and DCA^55^. TβMCA has been suggested as an antagonist of FXR^46^. Therefore, the increase in hepatic primary bile acids resulting from antibiotic administration is expected to affect FXR activity. Activation of FXR was reported to be involved in decreasing blood glucose and TG levels, liver regeneration, suppression of the expression of Cyp7a1 and Cyp51a1, and an increase in the expression of Cyp3A4/Cyp3a11^22,26,44,46,47^. The present results of liver weight loss, increased expression levels of Cyp7a1 and Cyp51a1, and decreased expression levels of Cyp3a11 by antibiotic administration may be at least partially explained by antagonism of FXR with an increasing hepatic level of TβMCA, while the decrease in the blood glucose and TG levels may be explained by agonism of FXR with a slight increase in the hepatic level of TCDCA. Thus, complex regulatory mechanisms involving multiple nuclear receptors are expected to be coordinate the effects of changes in secondary bile acids on the protein expression and physiology of the host. In the present report, proteomic analysis revealed that the expression levels of proteins involved in glucose and lipid metabolism changed in the mouse liver due to changes in secondary bile acids induced by antibiotic administration. However, the outputs derived from these results (e.g. changes in the amount of glycogen synthesis, adiponectin levels, the amount of bile acid synthesis, and the effect of primary bile acids) need to be evaluated by further studies.

In conclusion, the results of this study indicated that short-term decreases in hepatic secondary bile acid levels, from reductions in secondary bile acid-producing bacteria caused by antibiotic administration, led to decreases in serum TG and glucose levels and changes in the hepatic protein expression levels of Cyp2b10, Cyp3a25, and Cyp51a1 in mice. These results suggest that secondary bile acids produced by intestinal bacteria act as regulators for the homeostasis of glucose and TG levels and drug metabolism in the host. The present results also support the possibility that antibiotics that affect secondary bile acid-producing bacteria require caution in their use because of potential interactions with other drugs, including antidiabetic and antihyperlipidemic drugs and drugs metabolized by CYP2B6 or CYP3A4. Furthermore, secondary bile acid-producing bacteria have potential as a therapeutic or preventive target for metabolic diseases, such as diabetes and dyslipidemia.

## Materials and Methods

### Materials

Vancomycin hydrochloride, polymyxin B sulfate, lithocholic acid, sodium deoxycholate, sodium taurocholate, and lysyl endopeptidase were purchased from Wako Pure Chemical Industries (Osaka, Japan). QIAamp Fast DNA Stool Mini Kit was from Qiagen (Hilden, Germany). Taq DNA polymerase containing 10 × Standard buffer (Taq) and dNTPs were obtained from BioAcademia (Osaka, Japan) and synthesised PCR primers were obtained from FASMAC (Kanagawa, Japan). Sodium taurolithocholate and sodium taurochenodeoxycholate were purchased from Sigma-Aldrich (St. Louis, MO, USA). Taurodeoxycholic acid sodium salt was purchased from Nacalai Tesque (Kyoto, Japan). Tauro β-muricholic acid sodium salt was purchased from Steraloids (Newport, RI, USA). Taurocholic acid-d5 (TCA-d5) sodium salt was purchased from Toronto Research Chemicals (Toronto, Ontario, Canada). Triglyceride Quantification Assay kit was purchased from Abcam (Cambridge, UK). Plasma Membrane Protein Extraction Kit was obtained from BioVision (Milpitas, CA, USA). Pierce BCA Protein Assay Kit was obtained from Thermo Fisher Scientific (Waltham, MA, USA). Sequencing-grade modified trypsin (frozen) was obtained from Promega (Madison, WI, USA). Synthesised isotope-labelled peptides were obtained from Sigma-Aldrich. Other reagents were commercially available products of reagent or analytical grade.

### Treatment with antibiotics and secondary bile acids, and blood and tissue collection

Ten-week-old C57BL/6NSea male mice (Kyudo, Saga, Japan) were housed in individual cages with free access to food and water. VCM (500 mg/L) and PLB (100 mg/L) were dissolved in drinking water, and LCA (0.003% or 0.03% (wt/wt) of the diet) and DCA (0.05% or 0.1% (wt/wt) of the diet) were mixed with water and the mouse feed (CE-2 powder, CLEA Japan, Tokyo, Japan) and obtain a paste for 5 days. The paste was freshly prepared daily for 5 days. The liver and blood were collected (without food deprivation) at the end of treatment. The liver was weighed, frozen using liquid nitrogen, and stored at −80 °C. The faeces were collected immediately before (pre-administration) and at 5 days after administration to confirm the decrease in bacterial content in the faeces, and stored at −30 °C. All experiments on animals were approved by the Institutional Animal Care and Use Committee in Kumamoto University, and conducted in accordance with the regulations for animal experiments in Kumamoto University.

### Quantification of total bacteria and secondary bile acid-producing bacteria by PCR targeting the 16 S rRNA gene

DNA was extracted from a fixed amount of frozen faeces (200 mg) using the QIAamp Fast DNA Stool Mini Kit. The DNA extraction was conducted according to the manufacturer’s protocol with slight modifications. The homogenisation of stool samples was performed with a Micro Smash MS-100R (Tomy Seiko, Tokyo, Japan) with cooling, instead of a vortex mixer, to increase the recovery of DNA. The purity and yield of extracted DNA samples were confirmed from the absorbance at 260 and 280 nm. The extracted DNA solutions were diluted 100-fold with water, and the diluted DNA solutions from pre-administration faeces were further diluted to 50% and 25% (for total bacteria) or 12.5% (for *Clostridium* cluster XI and XIVa and *B*. *fragilis*) as quantitative references. Each PCR reaction mixture (20 μL total volume) consisted of 4 μL diluted DNA solution, 2 μL 10 × Standard buffer (Taq), 1.6 μL dNTPs (each 2.5 mM), 0.1 μL Taq DNA polymerase (5 unit/μL), 1 μL forward primer (10 μM), 1 μL reverse primer (10 μM), and water. The PCR amplification of the 16 S rRNA gene from total bacteria, *Clostridium* cluster XI and XIVa and *B*. *fragilis* was conducted using the primer sets as listed in Supplementary Table S3. After initial denaturation at 95 °C for 2 min, the reaction was subjected to cycles comprising denaturation at 95 °C for 30 s, annealing at 60 °C for 30 s, and elongation at 72 °C for 1 min, with a final elongation at 72 °C for 3 min. The UV transillumination of PCR products was detected by agarose gel electrophoresis.

### Quantification of bile acids in liver

The liver was homogenised in a 1- or 4-fold volume of water using a Shake Master NEO (Bio Medical Science, Tokyo, Japan) with cooling. Internal standard (TCA-d5, 10 ng), 50 μL of methanol, and 100 μL of acetonitrile were added to a portion of homogenate (100 μL) and mixed. The mixture was centrifuged at 21,600 × g for 10 min at 4 °C. The supernatant was mixed with 200 μL of 10 mmol/L ammonium acetate aqueous solution for LC−MS/MS analysis (the sample injection volume was 5 μL).

LC−MS/MS analysis was carried out with an HPLC system (Ultimate 3000, Thermo Fisher Scientific) connected to an Orbitrap Fusion Tribrid mass spectrometer (Thermo Fisher Scientific) equipped with a heated electrospray ionisation source in the negative-ion mode working in full-scan mode from *m/z* 300 to 700. Quantification of the target bile acids in the liver samples was conducted by the peak area of the accurate parent ions, and 482.2946, 498.2895, 498.2895, 514.2844, 514.2844, and 519.3158 were used as quantitative ions for TLCA, TDCA, TCDCA, TCA, TβMCA, and TCA-d5 (as internal standard), respectively. The analytes were separated at 40 °C with phenyl columns (ACQUITY UPLC BEH Phenyl, 2.1 mm ID × 150 mm, 1.7 μm particles, Waters, Milford, MA, USA). Linear gradients of acetonitrile in 10 mmol/L ammonium acetate aqueous solution were applied to elute the analytes at a flow rate of 300 μL/min for 25 min. For quantification of TLCA, TDCA, TCDCA, TCA and TβMCA, the respective calibration curve samples with concentrations in the 2.5–1000 ng/mL range were constructed with water as matrix.

### Biochemical blood tests and quantification of TG levels in liver

Blood was collected from the mice, incubated at 25 °C for 30 min and at 4 °C for 16 h, centrifuged at 1,000 × g for 30 min at 4 °C and the serum collected and stored at −80 °C until use. Biochemical blood tests were conducted with an automated analyser (Bio Majesty JCA-BM6050; JEOL, Tokyo, Japan). TG levels in the liver were quantified by colorimetric assay using the Triglyceride Quantification Assay kit according to the manufacturer’s protocol.

### Liver fractionation, protein quantification analysis, and functional annotation clustering and enrichment analysis

The fractionation of cytosol, crude membrane and plasma membrane, and the measurement of the protein concentration of the fractionated samples using the Plasma Membrane Protein Extraction Kit and the Pierce BCA Protein Assay Kit were performed according to the method described in a previous report^10^.

Protein expression levels were determined from five biological replicates by detection of specific peptides digested from the protein with sequential window acquisition of all theoretical fragment-ion spectra (SWATH) for focused quantitative proteomics, or with MRM/SRM for targeted quantitative proteomics. Peptide sample preparation and protein quantification analysis were conducted according to the method, and under the LC−MS/MS conditions, described previously^10,56^. The fractionated protein samples were denatured and solubilized in 12 mM sodium deoxycholate, 12 mM N-lauroylsarcosinate, and 100 mM Tris-HCl (pH 9.0). Then, the samples were reduced in 10 mM dithiothreitol, alkylated in 55 mM iodoacetamide, diluted 5-fold with 50 mM ammonium bicarbonate, and digested in lysyl endopeptidase for 3 hours at room temperature, and subsequently digested in sequence-grade modified trypsin for 16 hours at 37 °C. Stable isotope-labelled internal standard peptides were spiked into the peptide samples for targeted quantitative proteomics. The peptide samples were desalted for LC−MS analysis. In focused quantitative proteomics, the peptide sample of each group was analysed to obtain the MS/MS data from information-dependent acquisition, and the protein identification was conducted using ProteinPilot Software version 4.5 (Sciex, Framingham, MA, USA) with the Paragon algorithm and Uniprot mouse proteome database. The peaks of targeted peptides were extracted from the SWATH data using PeakView Software version 2.1 (Sciex) with the identified protein data. The sum of the peak-area values (intensities) of specific peptides from each protein was calculated as the protein expression level. In targeted quantitative proteomics, the precursor/product ion peaks from the targeted peptide and the internal standard peptide corresponding to the targeted peptide were detected with MRM/SRM. The intensities were calculated by Skyline version 3.1 (MacCoss Laboratory, University of Washington, Seattle, WA, USA), and the intensity ratios of the targeted peptide peak to the internal standard peptide peak for each product ion were calculated. The average value of the intensity ratios from four product ions was calculated as the protein expression level. The transition information for targeted quantitative proteomics (MRM/SRM measurement) is shown in Supplementary Table S4.

Functional annotation clustering and enrichment analysis from focused quantitative proteomics data was conducted using DAVID Bioinformatics Resources version 6.7 according to the method described in a previous report^10,57^. Enrichment terms in biological process category (GOTERM_BP_FAT) were identified using mouse whole genome background (DAVID default).

### Statistical analysis

The statistical significance of differences was determined by one-way ANOVA followed by Tukey’s test using Statistical Analysis System (SAS) software (Release 9.3, SAS Institute, Cary, NC, USA). For functional annotation clustering and enrichment analysis, the statistical significance of differences in protein expression levels from focused quantitative proteomics (AB mice vs. vehicle control mice and AB + SB (low) mice vs. AB mice) was determined by the Student’s t-test with Microsoft Excel version 14.0 (Microsoft, Redmond, WA, USA).

### Data availability statement

The datasets generated during and/or analysed during the current study are available from the corresponding author on reasonable request.

## Electronic supplementary material

Supplementary information
