# Supplementary material for: Reduction in hepatic secondary bile acids caused by short-term antibiotic-induced dysbiosis decreases mouse serum glucose and triglyceride levels
Source: Sci Rep. 2018 Jan 19;8:1253. doi: 10.1038/s41598-018-19545-1 (PMC5775293; doi:10.1038/s41598-018-19545-1)
Supplement: Supplementary file 1 — Supplementary information [file 41598_2018_19545_MOESM1_ESM.pdf]

Supplementary information

Reduction in hepatic secondary bile acids caused by short-term antibiotic-induced dysbiosis decreases mouse serum glucose and triglyceride levels

Takuya Kuno[1,2], Mio Hirayama-Kurogi[1,3], Shingo Ito[1,3,4], and Sumio Ohtsuki[*1,3,4]

[1]Department of Pharmaceutical Microbiology, Graduate School of Pharmaceutical Sciences, Kumamoto University, 5-1 Oe-honmachi, Chuo-ku, Kumamoto 862-0973, Japan

[2]Department of Drug Metabolism and Pharmacokinetics, Nonclinical Research Center, Tokushima Research Institute, Otsuka Pharmaceutical Co., Ltd., 463-10 Kagasuno, Kawauchi-cho, Tokushima, Tokushima 771-0192, Japan

[3]Department of Pharmaceutical Microbiology, Faculty of Life Sciences, Kumamoto University, 5-1 Oe-honmachi, Chuo-ku, Kumamoto 862-0973, Japan

[4]AMED-CREST, Japan Agency for Medical Research and Development, 1-7-1 Otemachi, Chiyoda, Tokyo 100-0004, Japan

[*]Correspondence and requests for materials should be addressed to S.O. (email: sohtsuki@kumamoto-u.ac.jp)

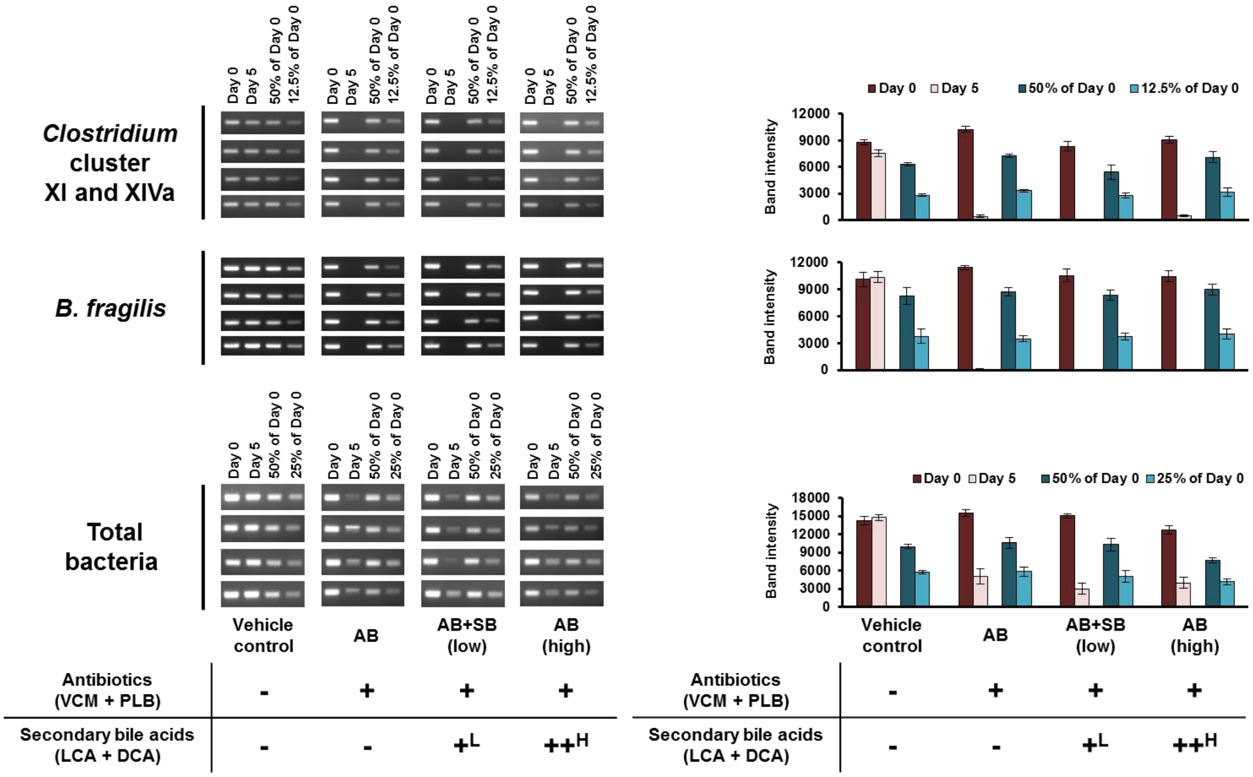

Supplementary Figure S1. PCR bands and intensities of bacterial 16S rRNA genes amplified from faeces collected from individual mice administered antibiotics with, or without, secondary bile acid supplementation

The PCR bands amplified from all of the individual mice, except those shown in Figure 1, are presented. The total bacterial content, including *Clostridium* clusters XI and XIVa, and *B. fragilis*, in faeces collected from mice administered antibiotics (VCM+PLB) with, or without, secondary bile acid (LCA+DCA) supplementation for 5 days was analysed by targeted PCR assays of the 16S rRNA gene for each bacterium. The faeces were collected pre-administration (Day 0) and at 5 days after administration (Day 5), and the templates extracted from a fixed amount of faeces (200 mg) were used in the PCR reactions. In Day 0 faeces, the PCR products amplified from diluted DNA samples [diluted to 50% and 25% (for total bacteria), and 12.5% (for *Clostridium* clusters XI and XIVa and *B. fragilis*)], were also analysed as quantitative references. Graphs on the right show the band intensities of PCR products as the mean ± SEM (n = 5, including the individuals in Figure 1). The intensities of PCR product bands were quantified using Image J software. ^{L}0.003% LCA and 0.05% DCA, and ^{H}0.03% LCA and 0.1% DCA, were mixed with the mouse feed.

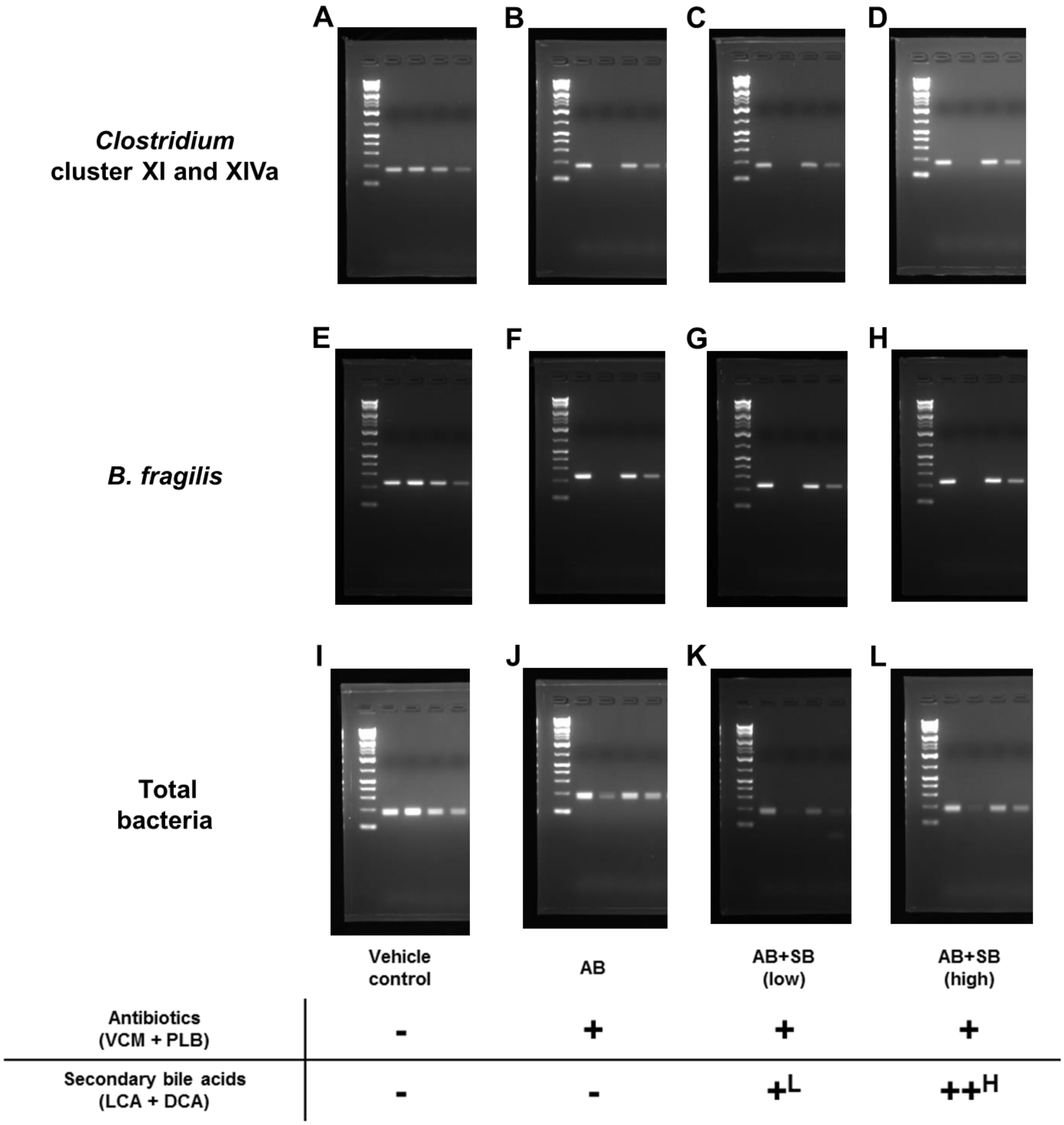

Supplementary Figure S2. Full-length gels

For *Clostridium* clusters XI and XIVa in feces collected from vehicle control (A), AB (B), AB+SB (low) (C), and AB+SB (high) (D) mice in Figure 1.

For *B. fragilis* in feces collected from vehicle control (E), AB (F), AB+SB (low) (G), and AB+SB (high) (H) mice in Figure 1.

For total bacteria in feces collected from vehicle control (I), AB (J), AB+SB (low) (K), and AB+SB (high) (L) mice in Figure 1.

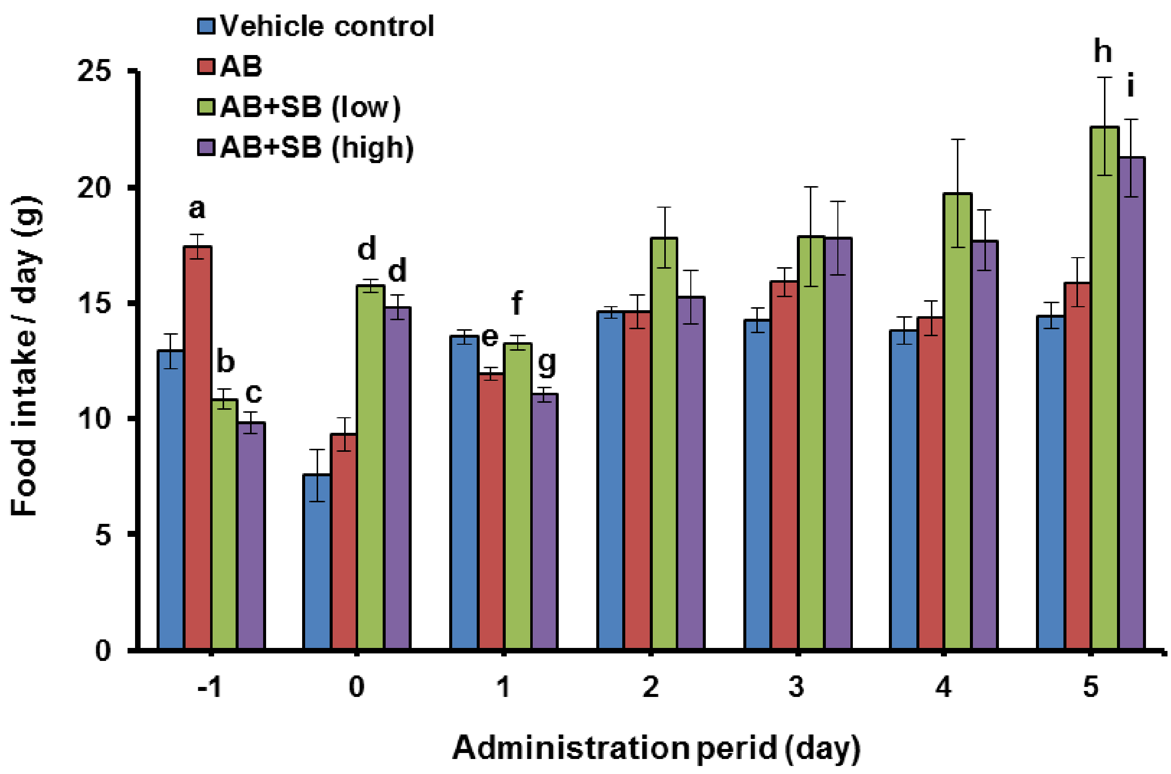

Supplementary Figure S3. Food intake in mice administered antibiotics with, or without, secondary bile acid supplementation

Data are presented as the mean ± SEM (n = 5). [a]Significantly different from vehicle control mice ($P < 0.001$). [b]Significantly different from AB mice ($P < 0.001$). [c]Significantly different from vehicle control ($P < 0.01$) and AB mice ($P < 0.001$). [d]Significantly different from vehicle control and AB mice ($P < 0.001$). [e]Significantly different from vehicle control mice ($P < 0.01$). [f]Significantly different from AB mice ($P < 0.05$). [g]Significantly different from vehicle control and AB+SB (low) mice ($P < 0.001$). [h]Significantly different from vehicle control ($P < 0.01$) and AB mice ($P < 0.05$). [i]Significantly different from vehicle control mice ($P < 0.05$). These food intake data include the amount of water evaporation from the paste used as feed and the amount of eating spillage from the mice.

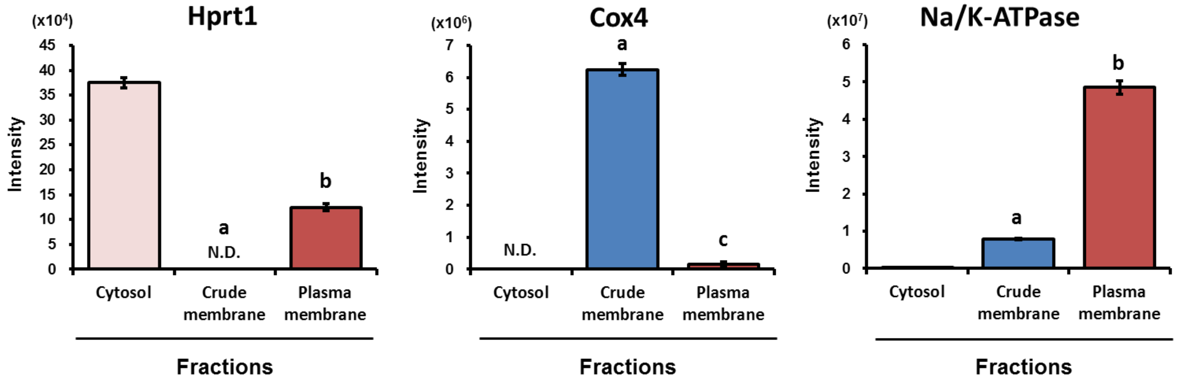

Supplementary Figure S4. Abundance of the marker proteins in the subcellular fractionated samples

The intensity of specific marker proteins to each fraction was calculated by focused quantitative proteomic analysis. Data are presented as the mean ± SEM (n = 20, all animals used in the present study). Hprt1, Cox4 (cytochrome c oxidase subunit 4, a marker of the mitochondrial inner membrane), and Na^+/K^+-ATPase were evaluated as marker proteins of the cytosol, crude membrane, and plasma membrane fractions, respectively. [a]Significantly different from cytosol fraction ($P < 0.001$). [b]Significantly different from cytosol and crude membrane fractions ($P < 0.001$). [c]Significantly different from crude membrane fraction ($P < 0.001$). N.D., not detected.

Supplementary Table S1. List of proteins that had significantly increased expression in AB mice compared with vehicle control mice, and had significantly decreased expression in AB+SB (low) mice compared with AB mice (P < 0.05)

< Liver cytosol fraction >

UniProt accession number	Protein name	Gene symbol	AB/ vehicle control (Fold change)	AB+SB (low)/ AB (Fold change)
tr|A2APN2|	Uridine phosphorylase 2	Upp2	2.79	0.23
sp|Q920E5|	Farnesyl pyrophosphate synthase	Fdps	2.74	0.35
tr|G3XA48|	Isopentenyl-diphosphate Delta-isomerase 1	Idi1	2.30	0.21
sp|Q8C0L9|	Glycerophosphocholine phosphodiesterase GPCPD1	Gpcpd1	1.96	0.52
tr|A2AQN4|	Acetyl-coenzyme A synthetase, cytoplasmic	Acss2	1.70	0.65
sp|Q8JZK9|	Hydroxymethylglutaryl-CoA synthase, cytoplasmic	Hmgcs1	1.61	0.50
sp|O55239|	Nicotinamide N-methyltransferase	Nnmt	1.61	0.61
sp|Q9WV68|	Peroxisomal 2,4-dienoyl-CoA reductase	Decr2	1.51	0.76
tr|F8VPN4|	Protein Agl	Agl	1.47	0.47
sp|Q62418|	Drebrin-like protein	Dbnl	1.32	0.78
sp|O35490|	Betaine--homocysteine S-methyltransferase 1	Bhmt	1.32	0.74
sp|P12970|	60S ribosomal protein L7a	Rpl7a	1.24	0.86
sp|P14148|	60S ribosomal protein L7	Rpl7	1.22	0.89
sp|Q9Z204|	Heterogeneous nuclear ribonucleoproteins C1/C2	Hnrnpc	1.22	0.82
sp|P14869|	60S acidic ribosomal protein P0	Rplp0	1.16	0.88
tr|Q922I7|	MCG13402, isoform CRA_c	Ptbp1	1.14	0.85
tr|A2ABY3|	Ethanolamine-phosphate cytidylyltransferase	Pcyt2	1.12	0.91

< Liver crude membrane fraction >

UniProt accession number	Protein name	Gene symbol	AB/ vehicle control (Fold change)	AB+SB (low)/ AB (Fold change)
sp|Q9D0E1|	Heterogeneous nuclear ribonucleoprotein M	Hnrnpm	2.59	0.52
sp|Q920E5|	Farnesyl pyrophosphate synthase	Fdps	2.26	0.44
tr|E9QJY0|	Low affinity cationic amino acid transporter 2	Slc7a2	2.14	0.37
tr|A2AA71|	Protein transport protein Sec24A	Sec24a	1.82	0.62
sp|Q9R1J0|	Sterol-4-alpha-carboxylate 3-dehydrogenase, decarboxylating	Nsdhl	1.82	0.38
sp|Q8K0C4|	Lanosterol 14-alpha demethylase	Cyp51a1	1.81	0.28
sp|O88455|	7-dehydrocholesterol reductase	Dhcr7	1.61	0.50
sp|O88736|	3-keto-steroid reductase	Hsd17b7	1.58	0.52
tr|Q9R1R8|	Retinol dehydrogenase 11	Rdh11	1.55	0.64
sp|O35490|	Betaine--homocysteine S-methyltransferase 1	Bhmt	1.52	0.85
sp|P50516|	V-type proton ATPase catalytic subunit A	Atp6v1a	1.44	0.55
sp|Q9WV54|	Acid ceramidase	Asah1	1.44	0.70
sp|Q9JIF7|	Coatomer subunit beta	Copb1	1.43	0.71
sp|P07724|	Serum albumin	Alb	1.42	0.67
sp|P11679|	Keratin, type II cytoskeletal 8	Krt8	1.40	0.80
sp|Q8K441|	ATP-binding cassette sub-family A member 6	Abca6	1.40	0.81
sp|Q76MZ3|	Serine/threonine-protein phosphatase 2A 65 kDa regulatory subunit A alpha isoform	Ppp2r1a	1.40	0.81
sp|Q71KT5|	Delta(14)-sterol reductase	Tm7sf2	1.40	0.62
sp|P34927|	Asialoglycoprotein receptor 1	Asgr1	1.39	0.79
tr|H7BWY6|	Retinol-binding protein 4	Rbp4	1.39	0.59
sp|P24721|	Asialoglycoprotein receptor 2	Asgr2	1.38	0.70
sp|O08677|	Isoform LMW of Kininogen-1	Kng1	1.38	0.82
sp|P28665|	Murinoglobulin-1	Mug1	1.34	0.71
sp|Q6PA06|	Atlastin-2	Atl2	1.34	0.73

sp|Q60605|	Myosin light polypeptide 6	Myl6	1.33	0.88
sp|Q08857|	Platelet glycoprotein 4	Cd36	1.32	0.78
sp|O70570|	Polymeric immunoglobulin receptor	Pigr	1.30	0.78
tr|Q569X9|	Cytochrome P450, family 2, subfamily c, polypeptide 67	Cyp2c67	1.29	0.75
sp|P70245|	3-beta-hydroxysteroid-Delta(8),Delta(7)-isomerase	Ebp	1.26	0.85
sp|O35604|	Niemann-Pick C1 protein	Npc1	1.25	0.78
sp|Q8VEK0|	Cell cycle control protein 50A	Tmem30a	1.24	0.83
sp|P21614|	Vitamin D-binding protein	Gc	1.24	0.79
sp|P14246|	Solute carrier family 2, facilitated glucose transporter member 2	Slc2a2	1.23	0.86
sp|P17047|	Lysosome-associated membrane glycoprotein 2	Lamp2	1.23	0.82
sp|Q9QZW0-	Isoform 2 of Probable phospholipid-transporting ATPase 11C	Atp11c	1.23	0.77
sp|Q9ERY9|	Probable ergosterol biosynthetic protein 28	ORF11	1.22	0.67
sp|Q68FD5|	Clathrin heavy chain 1	Cltc	1.22	0.77
sp|P12815|	Programmed cell death protein 6	Pdcd6	1.21	0.73
sp|P14094|	Sodium/potassium-transporting ATPase subunit beta-1	Atp1b1	1.21	0.82
sp|P21278|	Guanine nucleotide-binding protein subunit alpha-11	Gna11	1.20	0.78
sp|Q00897|	Alpha-1-antitrypsin 1-4	Serpina1d	1.20	0.74
sp|Q8VCH6|	Delta(24)-sterol reductase	Dhcr24	1.19	0.75
sp|O54749|	Cytochrome P450 2J5	Cyp2j5	1.18	0.81
sp|P11352|	Glutathione peroxidase 1	Gpx1	1.16	0.87
sp|Q05421|	Cytochrome P450 2E1	Cyp2e1	1.11	0.81
sp|Q9QUI0|	Transforming protein RhoA	Rhoa	1.10	0.92
sp|Q91V41|	Ras-related protein Rab-14	Rab14	1.09	0.87
sp|Q920L1|	Fatty acid desaturase 1	Fads1	1.09	0.82
tr|E0CXR0|	Bax inhibitor 1 (Fragment)	Tmbim6	1.07	0.85

< Liver plasma membrane fraction >

UniProt accession number	Protein name	Gene symbol	AB/ vehicle control (Fold change)	AB+SB (low)/ AB (Fold change)
sp|Q9WVM8|	Kynurenine/alpha-aminoadipate aminotransferase, mitochondrial	Aadat	2.85	0.59
tr|Q8R093|	Upp2 protein	Upp2	2.30	0.36
sp|Q920E5|	Farnesyl pyrophosphate synthase	Fdps	2.18	0.46
sp|Q8VCB3|	Glycogen [starch] synthase, liver	Gys2	2.06	0.38
tr|E9QJY0|	Low affinity cationic amino acid transporter 2	Slc7a2	1.88	0.57
sp|Q922Q8|	Leucine-rich repeat-containing protein 59	Lrrc59	1.84	0.67
sp|Q9WTP7|	GTP:AMP phosphotransferase AK3, mitochondrial	Ak3	1.70	0.65
sp|Q8C5H8|	NAD kinase 2, mitochondrial	Nadk2	1.70	0.76
sp|P51410|	60S ribosomal protein L9	Rpl9	1.58	0.54
tr|F8VPN4|	Protein Agl	Agl	1.58	0.56
sp|P20108|	Thioredoxin-dependent peroxide reductase, mitochondrial	Prdx3	1.57	0.61
sp|P85094|	Isochorismatase domain-containing protein 2A, mitochondrial	Isoc2a	1.55	0.78
sp|Q9ET01|	Glycogen phosphorylase, liver form	Pygl	1.54	0.57
sp|Q9D2G2|	Dihydrolipoyllysine-residue succinyltransferase component of 2-oxoglutarate dehydrogenase complex, mitochondrial	Dlst	1.52	0.80
tr|Q3UW66|	Sulfurtransferase	Mpst	1.51	0.72
sp|Q7TNG8|	Probable D-lactate dehydrogenase, mitochondrial	Ldhd	1.50	0.76
sp|P47738|	Aldehyde dehydrogenase, mitochondrial	Aldh2	1.49	0.75
sp|Q8BP67|	60S ribosomal protein L24	Rpl24	1.48	0.71
sp|P63276|	40S ribosomal protein S17	Rps17	1.44	0.82
sp|P62918|	60S ribosomal protein L8	Rpl8	1.43	0.73
tr|Q6ZWZ7|	60S ribosomal protein L17	Rpl17	1.39	0.74
sp|P62301|	40S ribosomal protein S13	Rps13	1.39	0.69
sp|Q60759|	Glutaryl-CoA dehydrogenase, mitochondrial	Gcdh	1.37	0.75
sp|P97807|	Fumarate hydratase, mitochondrial	Fh	1.36	0.83
sp|P09671|	Superoxide dismutase [Mn], mitochondrial	Sod2	1.33	0.78

sp|Q64433|	10 kDa heat shock protein, mitochondrial	Hspe1	1.33	0.77
sp|Q8VCW8|	Acyl-CoA synthetase family member 2, mitochondrial	Acsf2	1.32	0.75
sp|Q6ZWN5|	40S ribosomal protein S9	Rps9	1.32	0.77
tr|A2A547|	Ribosomal protein L19	Rpl19	1.31	0.73
tr|Q5XJF6|	Ribosomal protein	Rpl10a	1.31	0.73
sp|P62281|	40S ribosomal protein S11	Rps11	1.31	0.87
sp|Q9CR57|	60S ribosomal protein L14	Rpl14	1.31	0.77
sp|P14131|	40S ribosomal protein S16	Rps16	1.30	0.87
sp|P48776|	Tryptophan 2,3-dioxygenase	Tdo2	1.29	0.57
sp|P62702|	40S ribosomal protein S4, X isoform	Rps4x	1.28	0.80
sp|P62264|	40S ribosomal protein S14	Rps14	1.28	0.76
sp|P62855|	40S ribosomal protein S26	Rps26	1.25	0.82
sp|P62830|	60S ribosomal protein L23	Rpl23	1.19	0.82
sp|P14206|	40S ribosomal protein SA	Rpsa	1.19	0.85

Supplementary Table S2. List of proteins that had significantly decreased expression in AB mice compared with vehicle control mice, and had significantly increased expression in AB+SB (low) mice compared with AB mice (P < 0.05)

< Liver cytosol fraction >

UniProt accession number	Protein name	Gene symbol	AB/ vehicle control (Fold change)	AB+SB (low)/ AB (Fold change)
sp|Q64459|	Cytochrome P450 3A11	Cyp3a11	0.18	1.55
sp|Q8QZR3|	Pyrethroid hydrolase Ces2a	Ces2a	0.53	1.44
sp|Q64458|	Cytochrome P450 2C29	Cyp2c29	0.56	1.39
sp|Q91WG0|	Acylcarnitine hydrolase	Ces2c	0.63	1.39
sp|Q8R0W0|	Epiplakin	Eppk1	0.66	1.54
sp|Q8R146|	Acylamino-acid-releasing enzyme	Apeh	0.72	1.26
sp|Q9EQ06|	Estradiol 17-beta-dehydrogenase 11	Hsd17b11	0.73	1.72
sp|Q5SWU9|	Acetyl-CoA carboxylase 1	Acaca	0.75	1.17
sp|Q8VCR2|	Isoform 2 of 17-beta-hydroxysteroid dehydrogenase 13	Hsd17b13	0.76	1.70
sp|P33267|	Cytochrome P450 2F2	Cyp2f2	0.77	1.44
sp|Q8VC30|	Bifunctional ATP-dependent dihydroxyacetone kinase/FAD-AMP lyase (cyclizing)	Dak	0.77	1.13
sp|Q9JMH6|	Thioredoxin reductase 1, cytoplasmic	Txnrd1	0.78	1.16
tr|Q3UWB9|	UDP-glucuronosyltransferase 2B17	Ugt2b5	0.79	1.23
tr|E9Q1Q9|	Ketohexokinase	Khk	0.81	1.15
sp|Q8BVI4|	Dihydropteridine reductase	Qdpr	0.82	1.23
sp|P40936|	Indolethylamine N-methyltransferase	Inmt	0.85	1.27
sp|P16858|	Glyceraldehyde-3-phosphate dehydrogenase	Gapdh	0.89	1.09
tr|Q3UEL5|	Urocanate hydratase	Uroc1	0.89	1.13
sp|P10605|	Cathepsin B	Ctsb	0.93	1.08

< Liver crude membrane fraction >

UniProt accession number	Protein name	Gene symbol	AB/ vehicle control (Fold change)	AB+SB (low)/ AB (Fold change)
sp|Q64459|	Cytochrome P450 3A11	Cyp3a11	0.11	1.54
tr|F8VPN4|	Protein Agl	Agl	0.18	8.59
sp|Q9ET01|	Glycogen phosphorylase, liver form	Pygl	0.25	6.34
sp|Q5RKZ7|	Molybdenum cofactor biosynthesis protein 1	Mocs1	0.41	2.60
sp|Q8VCB3|	Glycogen [starch] synthase, liver	Gys2	0.41	7.15
sp|Q5YD48|	APOBEC1 complementation factor	A1cf	0.42	1.79
sp|Q9CQU0|	Thioredoxin domain-containing protein 12	Txndc12	0.46	2.54
sp|O09158|	Cytochrome P450 3A25	Cyp3a25	0.61	1.44

sp|Q8C7E7|	Starch-binding domain-containing protein 1	Stbd1	0.67	1.34
sp|Q2TPA8|	Hydroxysteroid dehydrogenase-like protein 2	Hsdl2	0.74	1.25
sp|Q9DBM2|	Peroxisomal bifunctional enzyme	Ehhadh	0.82	1.25
sp|Q9DCS3|	Trans-2-enoyl-CoA reductase, mitochondrial	Mecr	0.82	1.26
tr|Q3TJI8|	Corticosteroid 11-beta-dehydrogenase isozyme 1	Hsd11b1	0.85	1.14
sp|Q8BU33|	Acetolactate synthase-like protein	Ilvbl	0.87	1.12
sp|Q6PB66|	Leucine-rich PPR motif-containing protein, mitochondrial	Lrpprc	0.89	1.16
sp|Q8CIM7|	Cytochrome P450 2D26	Cyp2d26	0.89	1.23

< Liver plasma membrane fraction >

UniProt accession number	Protein name	Gene symbol	AB/ vehicle control (Fold change)	AB+SB (low)/ AB (Fold change)
sp|Q04736|	Tyrosine-protein kinase Yes	Yes1	0.32	1.89
sp|Q05117|	Tartrate-resistant acid phosphatase type 5	Acp5	0.36	1.86
sp|Q7TMS5|	ATP-binding cassette sub-family G member 2	Abcg2	0.49	1.54
tr|Q32ME1|	ATPase, Ca++ transporting, plasma membrane 4	Atp2b4	0.51	2.28
sp|B2RX12|	Isoform 3 of Canalicular multispecific organic anion transporter 2	Abcc3	0.53	1.45
sp|O08966|	Solute carrier family 22 member 1	Slc22a1	0.53	1.32
sp|P97384|	Annexin A11	Anxa11	0.61	1.40
sp|P24472|	Glutathione S-transferase A4	Gsta4	0.61	1.60
sp|Q9JJ00|	Phospholipid scramblase 1	Plscr1	0.62	1.90
sp|Q9EPK2|	Protein XRP2	Rp2	0.64	1.31
sp|Q9JJJ3|	Aquaporin-9	Aqp9	0.65	1.19
sp|P16406|	Glutamyl aminopeptidase	Enpep	0.66	1.54
sp|P62874|	Guanine nucleotide-binding protein G(I)/G(S)/G(T) subunit beta-1	Gnb1	0.69	1.28
sp|Q9DAS9|	Guanine nucleotide-binding protein G(I)/G(S)/G(O) subunit gamma-12	Gng12	0.71	1.16
sp|P27601|	Guanine nucleotide-binding protein subunit alpha-13	Gna13	0.72	1.53
tr|E9QKR0|	Guanine nucleotide-binding protein G(I)/G(S)/G(T) subunit beta-2	Gnb2	0.73	1.30
sp|P80316|	T-complex protein 1 subunit epsilon	Cct5	0.74	1.37
sp|P97429|	Annexin A4	Anxa4	0.74	1.39
tr|Q9D3L3|	Synaptosomal-associated protein	Snap23	0.74	1.31
sp|P21278|	Guanine nucleotide-binding protein subunit alpha-11	Gna11	0.75	1.25
sp|Q60770|	Syntaxin-binding protein 3	Stxbp3	0.76	1.17
sp|P11983|	T-complex protein 1 subunit alpha	Tcp1	0.76	1.24
sp|P80317|	T-complex protein 1 subunit zeta	Cct6a	0.76	1.28
tr|B1AX58|	Plastin-3	Pls3	0.78	1.26
sp|P40936|	Indolethylamine N-methyltransferase	Inmt	0.79	1.29
sp|Q80SZ7|	Guanine nucleotide-binding protein G(I)/G(S)/G(O) subunit gamma-5	Gng5	0.80	1.27
sp|P80314|	T-complex protein 1 subunit beta	Cct2	0.80	1.18
sp|P80315|	T-complex protein 1 subunit delta	Cct4	0.81	1.26
sp|P80318|	T-complex protein 1 subunit gamma	Cct3	0.81	1.20
sp|P97792|	Coxsackievirus and adenovirus receptor homolog	Cxadr	0.81	1.38
sp|P08752|	Guanine nucleotide-binding protein G(i) subunit alpha-2	Gnai2	0.84	1.28
sp|Q61081|	Hsp90 co-chaperone Cdc37	Cdc37	0.85	1.24

Supplementary Table S3. Primer sequences used for PCR

Target	Primer	Sequence (5'-3')	Amplicon size (bp)	PCR cycle	Reference
All bacteria	Eub338F	ACTCCTACGGGAGGCAGCAG	200	21	1
	Eub518R	ATTACCGCGGCTGCTGG			
clostridium cluster	Erec 688F	GCGTAGATATTAGGAGGAAC	172	23	2
XI and XIVa	Erec 841R	TGCGTTWGCKRCGGCACCG			
B. fragilis	Bfr-F	CTGAACCAGCCAAGTAGCG	230	28	3
	Bfr-R	CCGCAAACTTTCACAACTGACTTA			

Supplementary Table S4. Transition information for MRM/SRM measurements

Protein	Peptide sequence	Unlabelled peptide					Internal standard labelled peptide				
		Q1	Q3-1	Q3-2	Q3-3	Q3-4	Q1	Q3-1	Q3-2	Q3-3	Q3-4
Cyp2b10	GTVAVVEPTFK	574.3	492.3	720.4	819.5	890.5	578.3	500.3	728.4	827.5	898.5
Cyp3a11	LYPIANR	423.7	277.2	285.7	473.3	570.3	428.7	277.2	290.7	483.3	580.3
Cyp3a25	TLLSPTFTSGK	576.3	215.1	737.4	824.4	937.5	580.3	215.1	745.4	832.4	945.5
Cyp51a1	FAYVPFGAGR	542.8	219.1	382.2	481.2	604.3	547.8	219.1	382.2	481.2	614.3
Na/K-ATPase	IVEIPFNSTNK	613.3	213.2	342.2	807.4	1049.5	635.4	213.2	342.2	815.4	1057.5

Underlining indicates the position of the amino acid labelled with stable isotopes.

REFERENCES

1. Ferrand, J. *et al.* Comparison of seven methods for extraction of bacterial DNA from fecal and cecal samples of mice. *J. Microbiol. Methods* **105,** 180–185 (2014).
2. Vanhoutte, T. *et al.* Molecular monitoring of the fecal microbiota of healthy human subjects during administration of lactulose and Saccharomyces boulardii. *Appl. Environ. Microbiol.* **72,** 5990–5997 (2006).
3. Liu, C. *et al.* Rapid identification of the species of the Bacteroides fragilis group by multiplex PCR assays using group-and species-specific primers. *FEMS Microbiol. Lett.* **222,** 9–16 (2003).
